# In vitro and in silico characterization of adiponectin-receptor agonist dipeptides

**DOI:** 10.1038/s41538-021-00114-2

**Published:** 2021-11-12

**Authors:** Yuna Lee, Akihiro Nakano, Saya Nakamura, Kenta Sakai, Mitsuru Tanaka, Keisuke Sanematsu, Noriatsu Shigemura, Toshiro Matsui

**Affiliations:** 1 Department of Bioresources and Biosciences, Faculty of Agriculture, Graduate School of Kyushu University, 744 Motooka, Nishi-ku, Fukuoka, 819-0395 Japan; 2 Research and Development Center for Five-Sense Devices, Kyushu University, 744 Motooka, Nishi-ku, Fukuoka, 819-0395 Japan; 3 Section of Oral Neuroscience, Graduate School of Dental Science, Kyushu University, 3-1-1 Maidashi, Higashi-ku, Fukuoka, 812-8582 Japan; 4 Oral Health/Brain Health/Total Health Research Center, Graduate School of Dental Science, Kyushu University, 3-1-1 Maidashi, Higashi-ku, Fukuoka, 812-8582 Japan

**Keywords:** Nutrition, Peptides

## Abstract

The aim of this study is to develop a dipeptide showing an adiponectin receptor 1 (AdipoR1) agonistic effect in skeletal muscle L6 myotubes. Based on the structure of the AdipoR1 agonist, AdipoRon, 15 synthetic dipeptides were targeted to promote glucose uptake in L6 myotubes. Tyr-Pro showed a significant increase in glucose uptake among the dipeptides, while other dipeptides, including Pro-Tyr, failed to exert this effect. Tyr-Pro induces glucose transporter 4 (Glut4) expression in the plasma membrane, along with adenosine monophosphate-activated protein kinase (AMPK) activation. In AdipoR1-knocked down cells, the promotion by Tyr-Pro was ameliorated, indicating that Tyr-Pro may directly interact with AdipoR1 as an agonist, followed by the activation of AMPK/Glut4 translocation in L6 myotubes. Molecular dynamics simulations revealed that a Tyr-Pro molecule was stably positioned in the two potential binding pockets (sites 1 and 2) of the seven-transmembrane receptor, AdipoR1, anchored in a virtual 1-palmitoyl-2-oleoyl-phosphatidylcholine membrane. In conclusion, we demonstrated the antidiabetic function of the Tyr-Pro dipeptide as a possible AdipoR1 agonist.

## Introduction

Appropriate blood glucose control may help prevent the increasing onset of non-insulin-dependent type II diabetes (NIDDM)^1^. Thus, alternative medicinal food intake, similar to a daily intake of acarbose, an anti-hyperglycemic drug^2^; promoting glucose availability in exercise would be acceptable for appropriate management of blood glucose levels at early stages of pre-diabetes. In our previous report using spontaneously diabetic Torii rats (SDT), daily intake of a rose hip extract^3^ suppressed the onset of diabetes by ameliorating impaired glucose tolerance. Studies of alternative medicinal foods have demonstrated possible improvement of impaired glucose availability by stimulating adenosine monophosphate-activated protein kinase (AMPK)-mediated glucose transporter 4 (Glut4) translocation pathways independent of the insulin/phosphatidylinositol 3-kinase (PI3K)/Akt-mediated Glut4 translocation pathways^4,5^. In rat skeletal muscle L6 cell experiments, theasinensins, condensed catechins, promoted glucose uptake through the calcium-calmodulin-dependent protein kinase kinase (CaMKK)/AMPK-mediated Glut4 translocation pathway^4^. The dipeptide Trp-His also promoted glucose uptake in L6 myotubes via the peptide/histidine transporter 1 (PHT1)/liver kinase B1 (LKB1)/AMPK/Glut4 pathway^5^. Besides those, other different bioactive peptides, such as cereal proteins-derived peptides, IQP, IPQ, VPE, and VEP^6^, and milk casein hydrolysate-derived peptide, IPP^7^, have been revealed to promote glucose uptake via the activation of AMPK. Therefore, activating non-insulin AMPK signaling pathways by any bioactive compound may be beneficial for preventing NIDDM in an insulin-independent manner.

**Fig. 1:**
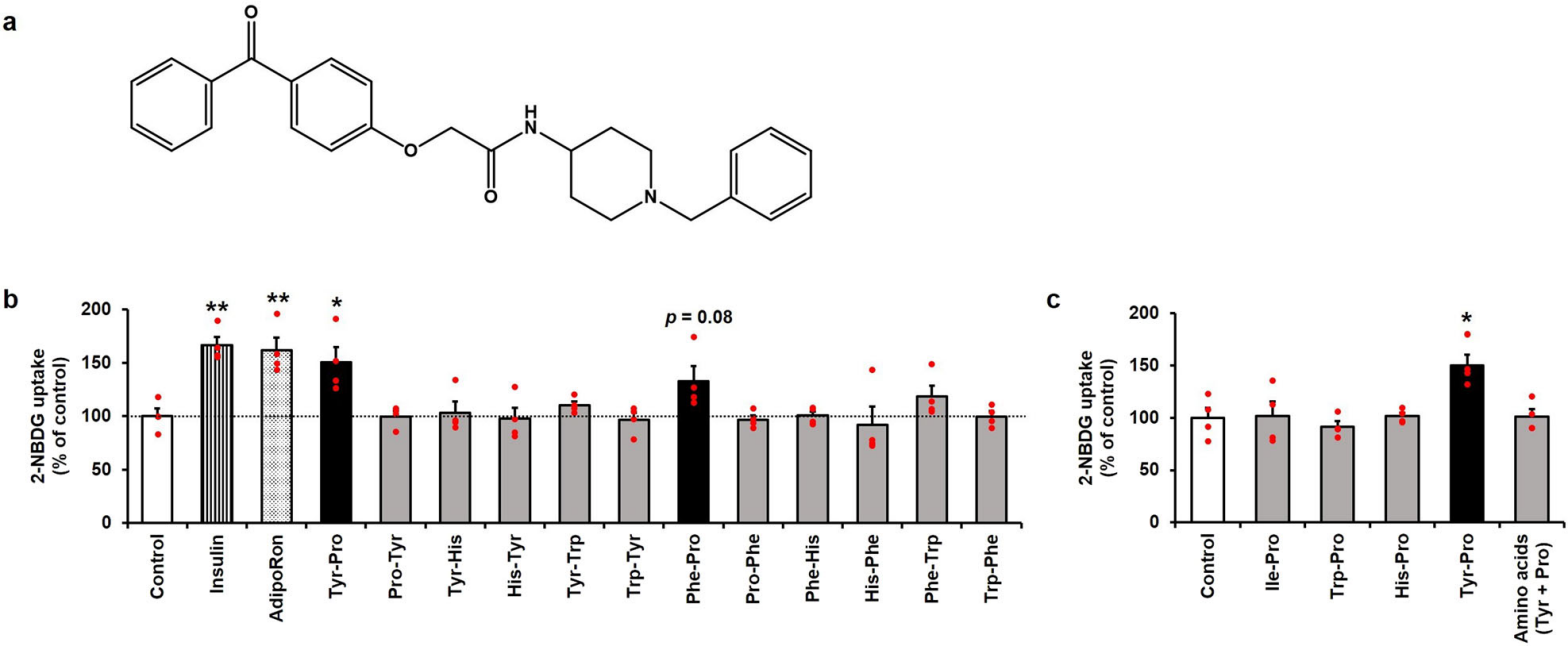
Screening for dipeptides that promote 2-NBDG uptake in L6 myotubes. **a** Chemical structure of AdipoRon. **b** 2-NBDG uptake by 12 dipeptides (Tyr-Pro, Pro-Tyr, Tyr-His, His-Tyr, Tyr-Trp, Trp-Tyr, Phe-Pro, Pro-Phe, Phe-His, His-Phe, Phe-Trp, and Trp-Phe). 2-NBDG uptake experiments of 10 µM dipeptides for 0.5 h in L6 myotubes were performed. Insulin (0.1 µM) and AdipoRon (0.1 µM) were used as positive control. **c** 2-NBDG uptake by four dipeptides containing Pro at the C-terminus (Ile-Pro, Trp-Pro, His-Pro, and Tyr-Pro). L6 myotubes were incubated with 10 µM dipeptides or a mixture of Tyr and Pro (Tyr + Pro) for 0.5 h. The 2-NBDG incorporated in L6 myotubes was assayed at excitation and emission wavelengths of 465 and 540 nm, respectively. Results are expressed as the mean ± SEM (*n* = 4). Statistical differences were evaluated using unpaired two-tailed Student’s *t*-test. *p < 0.05, **p < 0.01 vs. control.

Stimulation of adiponectin receptors (AdipoR) promotes glucose uptake via an insulin-independent pathway^6^. AdipoR may be a preferable AMPK-dependent Glut4 translocation cascade. Therefore, AdipoR stimulation can be suggested as the replacement therapy of adiponectin against the NIDDM because an adiponectin-like therapeutic agent, AdipoRon (2-(4-benzoylphenoxy)-*N*-[1-(phenylmethyl)-4-piperidinyl]acetamide) (Fig. 1a), an orally active synthetic small-molecule AdipoR agonist, showed in vivo evidence that impaired glucose tolerance and insulin resistance in *db*/*db* mice with reduced high-fat diet-induced adiponectin secretion was improved by AdipoRon (50 mg/kg)^7^. Recent reports also claimed in vivo biological effectiveness of AdipoRon in restoring renal AdipoR expression^8^ and improved vascular dysfunction in diabetic *db*/*db* mice^9^. Although the evidence led us to investigate a strategy for modulation or prevention of NIDDM with obesity-related glucose tolerance by adiponectin-like food compounds, no alternative-medicinal food studies regarding an adiponectin-like “orally active” natural agonist have been reported so far.

Adiponectin, an adipocyte-derived hormone^10^, directly binds to two distinct seven-transmembrane receptors, AdipoR1 and R2^11^, which are mainly expressed in skeletal muscles and in the liver, respectively^12^. These receptors can activate multiple signaling molecules, such as AMPK, p38 mitogen-activated protein kinase (MAPK), and peroxisome proliferator-activated receptor α (PPARα), which are activated by adiponectin in AdipoR-overexpressing skeletal muscle cells, following the promotion of glucose uptake^12,13^. Thus far, it has been reported that the intracellular signaling axis of the adiponectin hormone towards glucose uptake in skeletal muscles mainly involves the AdipoR1-mediated AMPK/Glut4 translocation cascade^6,14,15^. Moreover, the adiponectin-induced activation of AMPK also plays an important role in the upregulation of mitochondrial function^16^. Therefore, it is speculated that food compounds showing AdipoR agonistic effects, such as adiponectin, are good candidates for alternative prevention strategies in diabetes with impaired adiponectin secretion and insulin resistance.

In this study, we attempted to discover AdipoR1 agonistic dipeptides, since the structural features of AdipoRon with characteristic 1-benzyl 4-substituted 6-membered cyclic amine moieties and aromatic rings linked with amide bonds (Fig. 1a)^7^ can be mimicked by a peptide skeleton-bearing aromatic rings and peptide bonds. In a previous report^17^, 15-mer oligopeptides designed by in silico AdipoR1 protein–ligand docking simulation of the crystal structure were confirmed to activate AdipoR1-mediated signaling in mouse skeletal muscle cells. Although the report revealed the possible development of a peptide-derived AdipoR1 agonist, our in vitro study on AdipoR1 agonistic dipeptides in rat skeletal muscle L6 cells is distinct from the report by Kim et al.^17^. In our series of studies on the bioavailability of peptides^18–22^, dipeptides are preferably absorbed into the blood system in intact form, while no evidence of intact absorption of longer oligopeptides, including 15-mer oligopeptides, has been reported. The advantage of dipeptides may thus allow the development of “orally active” functional foods exerting AdipoR1-mediated prevention of NIDDM. Another novelty of this study lies in the in silico molecular dynamics (MD) simulation using the CHARMM-GUI, in which membrane-bound AdipoR1 is virtually anchored in the phospholipid membrane, and molecular interaction of AdipoR1 agonist dipeptides with the AdipoR1 protein could be validated in virtual physiological environments.

## Results

### Screening of dipeptides promoting glucose uptake in L6 myotubes

AdipoRon, an AdipoR agonist, was designed to exert an adiponectin-like effect by its characteristic 1-benzyl 4-substituted six-membered cyclic amine moiety and aromatic rings linked with amide bonds (Fig. 1)^7^. Referring to the active structural information of AdipoRon for AdipoR1 binding, we synthesized 12 aromatic dipeptides (Tyr-Pro, Pro-Tyr, Tyr-His, His-Tyr, Tyr-Trp, Trp-Tyr, Phe-Pro, Pro-Phe, Phe-His, His-Phe, Phe-Trp, and Trp-Phe). The synthetic dipeptides were subjected to glucose (or 2-NBDG) uptake experiments in rat skeletal muscle L6 myotubes at a concentration of 10 µM. Two dipeptides, Tyr-Pro (150 ± 14%, *p* < 0.05) and Phe-Pro (133 ± 14%, *p* = 0.08), were candidates for promoting 2-NBDG uptake in L6 myotubes, similar to insulin (0.1 µM, 166 ± 8%) and AdipoRon (0.1 µM, 162 ± 12%) under the present cellular conditions. In contrast, Pro-Tyr and Pro-Phe with reversed sequences of the above two active peptides failed to promote the uptake (Fig. 1b). This observation led us to strongly consider the importance of the peptide sequence and the imino group from Pro positioned at the C-terminus in exerting a glucose-promoting effect in L6 myotubes. Hence, four C-terminal Pro-dipeptides (Ile-Pro, Trp-Pro, His-Pro, and Tyr-Pro) were newly targeted for further screening of 2-NBDG-promoting dipeptides in L6 myotubes. As shown in Fig. 1c, no significant promoting effect of Pro-dipeptides, except for Tyr-Pro (*p* < 0.05), was observed, suggesting that aromatic amino acids at the N-terminus of Pro-dipeptides are not a determining factor for this effect. Configurational fitting of Tyr-Pro with target signals or receptors in L6 cells may be associated with this effect. The lack of increased 2-NBDG uptake by a mixture of Tyr and Pro (Fig. 1c) strongly indicates the importance of the peptide bond between Tyr and Pro (or the carbonyl group and tertiary amine) for glucose promotion.

### Promotion of glucose uptake by Tyr-Pro in L6 myotubes

**Fig. 2:**
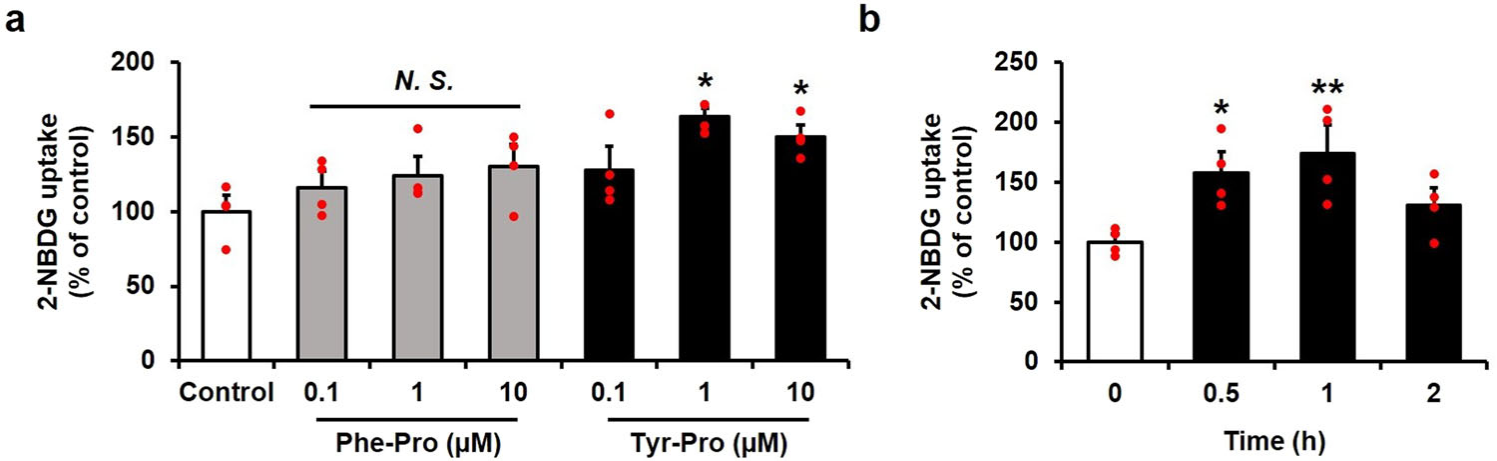
2-NBDG uptake by dipeptides in L6 myotubes. **a** The effect of Phe-Pro or Tyr-Pro concentrations on 2-NBDG uptake. L6 myotubes were incubated with 0.1, 1, or 10 µM dipeptides for 0.5 h. **b** The effect of incubation time with 1 µM Tyr-Pro on 2-NBDG uptake. L6 myotubes were incubated for 0.5, 1, or 2 h. Results are expressed as the mean ± SEM (*n* = 4). Statistical differences were evaluated using Dunnett’s *t*-test. *p < 0.05, **p < 0.01 vs. control; N.S. no significant difference at *p* > 0.05.

Next, we investigated the potential of Tyr-Pro to promote 2-NBDG uptake in L6 myotubes. The effect of Tyr-Pro on 2-NBDG uptake was examined as a function of concentration (0.1, 1, or 10 µM) or incubation time (0.5, 1, or 2 h). Under the experimental conditions, no Tyr–Pro cytotoxicity was found in L6 myotubes (Fig. S1). As shown in Fig. 2a, a significant (*p* < 0.05) promoting response of Tyr-Pro was obtained at a concentration of more than 1 µM when L6 myotubes were treated for 0.5 h. In contrast, Phe-Pro, which tended to promote the uptake at 10 µM (Fig. 1b), failed to promote uptake at lower concentrations (Fig. 2a), suggesting that the dipeptide was categorized as a weak glucose promoter. The promoting effect of Tyr-Pro was time-dependent, achieving the maximal promoting effect at 1 h (174 ± 23%) (Fig. 2b). Further experiments of active Tyr-Pro were performed at 1 µM concentration for 1 h.

### Involvement of AdipoR1 in Tyr-Pro-induced glucose uptake in L6 myotubes

**Fig. 3:**
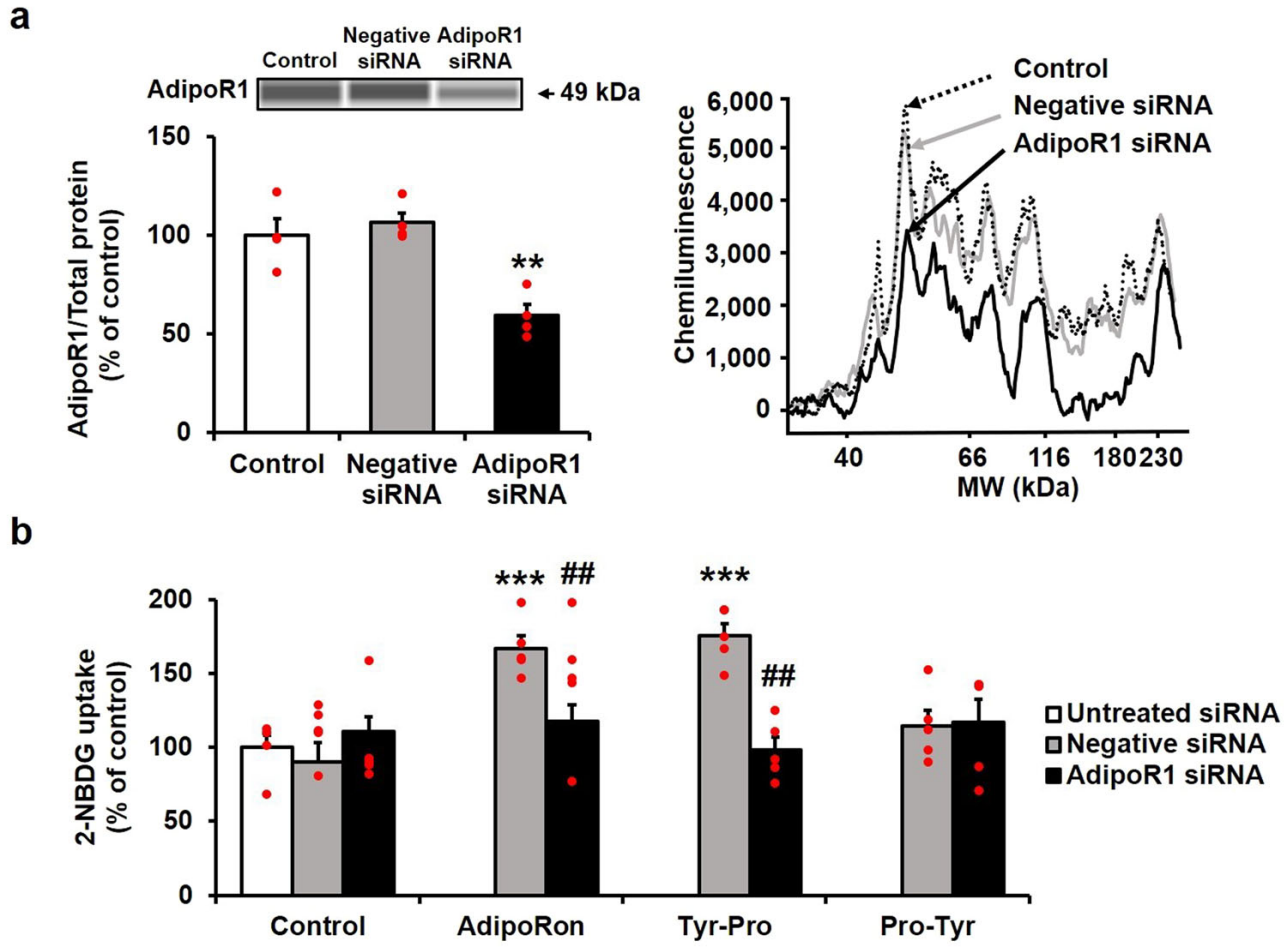
Involvement of the AdipoR1 receptor in promoting 2-NBDG uptake in L6 myotubes by Tyr-Pro. **a** AdipoR1-knockdown of L6 myotubes by AdipoR1-specific siRNA transfection. The AdipoR1 protein level was measured using the Wes instrument. The chemiluminescent signal is displayed as a virtual blot-like image, and an electropherogram is generated based on molecular weights. **b** The effect of AdipoR1-knockdown in L6 myotubes on the promotion of 2-NBDG uptake by dipeptides. The knocked-down L6 myotubes were incubated with 1 µM Tyr-Pro or 1 µM Pro-Tyr for 1 h for 2-NBDG uptake experiments. AdipoRon (0.1 µM, 0.5 h) was used as a positive control to validate the knockdown of AdipoR1 in L6 myotubes. Results are expressed as the mean ± SEM (*n* = 4–5). Statistical differences were evaluated using Dunnett’s *t*-test. **p < 0.01, ***p < 0.001 vs. control. Statistical differences between negative and AdipoR1 siRNA groups were evaluated by unpaired two-tailed Student’s *t*-test. ^##^*p* < 0.01.

The involvement of AdipoR1 in promoting 2-NBDG uptake by AdipoRon-mimicking Tyr-Pro (Fig. 1b) was examined in AdipoR1-knocked down L6 myotubes. The AdipoR1-knockdown of L6 myotubes by AdipoR1-specific siRNA transfection (Figs. 3a and S2) resulted in a significant (*p* < 0.05) disappearance of Tyr-Pro-induced promotion of 2-NBDG uptake (Fig. 3b). A similar reduction in uptake in the knockdown cells was also observed for the AdipoR1-agonist AdipoRon (Fig. 3b), indicating that the promotion in L6 myotubes by both AdipoRon and Tyr-Pro was mediated by AdipoR1. The observed Tyr-Pro sequence-specific promotion via AdipoR1 recognition was also validated by the lack of change in the glucose uptake of Trp-His, a PHT1 (but not AdipoR1)-mediated promoter of glucose uptake^5^ in AdipoR1-knocked down L6 myotubes (Fig. S3).

### Effect of Tyr-Pro on Glut4 translocation in L6 myotubes

**Fig. 4:**
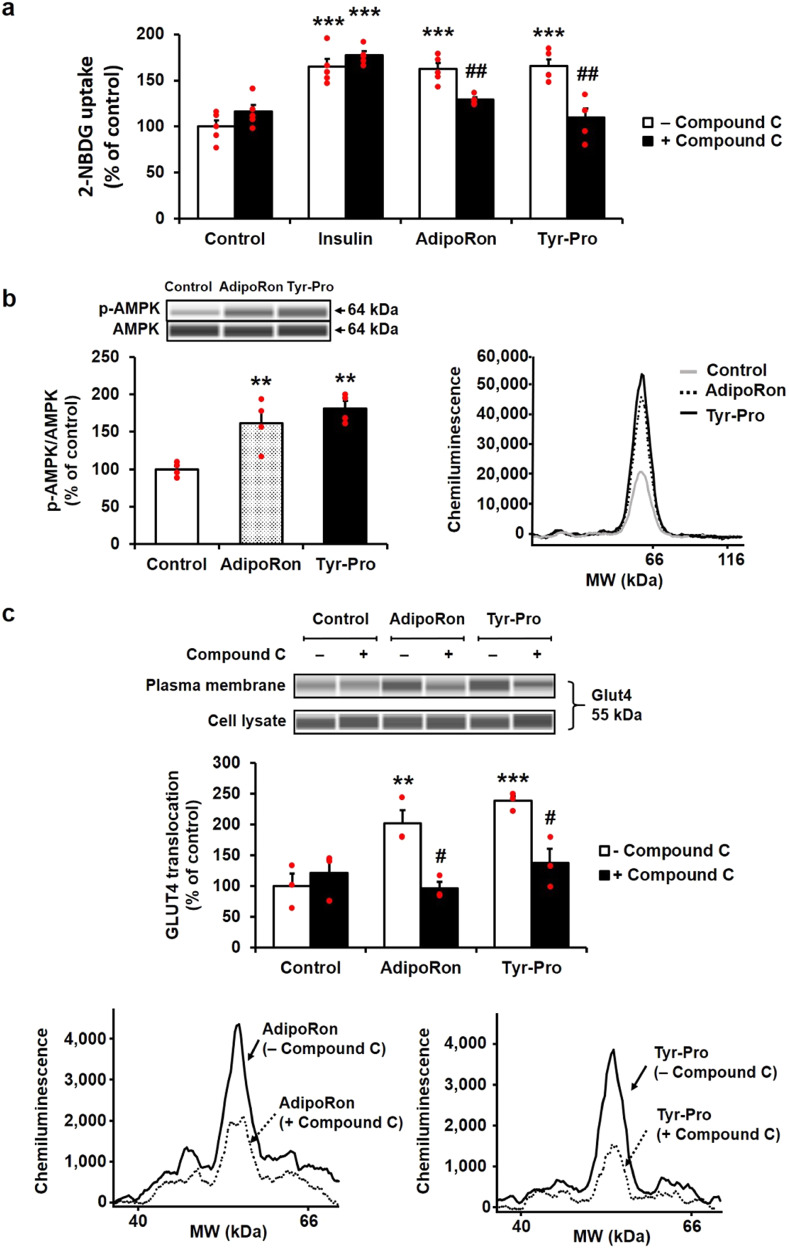
The involvement of AMPK activation in Tyr-Pro-induced promotion of 2-NBDG uptake in L6 myotubes. **a** The effect of compound C (an AMPK inhibitor) on the promotion of 2-NBDG uptake by Tyr-Pro. L6 myotubes were incubated with 1 µM Tyr-Pro for 1 h in the presence or absence of 20 µM compound C. Insulin (0.1 µM, 0.5 h) was used as negative control (AMPK-independent uptake), while AdipoRon (0.1 µM, 0.5 h) was used as a positive control (AdipoR1-mediated AMPK-dependent uptake). The effect of Tyr-Pro on AMPK activation (**b**) and Glut4 expression (**c**) in L6 myotubes was measured using the Wes instrument. p-AMPK/AMPK in cell lysates and Glut4 protein expression in PM and cell lysates were evaluated in L6 myotubes treated with 1 µM Tyr-Pro for 1 h. Results are expressed as the mean ± SEM (*n* = 3–5). Statistical differences were evaluated using Dunnett’s *t*-test. **p < 0.01 vs. control, ***p < 0.001 vs. control. Statistical differences between two groups in the presence and absence of compound C were evaluated by unpaired two-tailed Student’s *t*-test. ^#^*p* < 0.05, ^##^*p* < 0.01.

To clarify the mechanism of the promotion of glucose uptake by Tyr-Pro, the Glut4 translocation cascade in L6 myotubes was examined. As shown in Fig. 4, the AMPK signaling inhibition by compound C, a specific AMPK inhibitor, abolished (*p* < 0.05) the promotion of 2-NBDG uptake by Tyr-Pro, similar to AdipoRon. AMPK activation in L6 myotubes treated with Tyr-Pro and AdipoRon was also confirmed by an increase in the phosphorylation level of AMPK (Figs. 4b and S2). As shown in Figs. 4c and S2, Glut4 expression in the plasma membrane of L6 myotubes was promoted by Tyr-Pro, similar to AdipoRon, indicating that Tyr-Pro may stimulate the Glut4 translocation cascade through the activation of AdipoR1/AMPK signaling.

### In silico simulation analysis of the Tyr-Pro–AdipoR1 docking complex

**Fig. 5:**
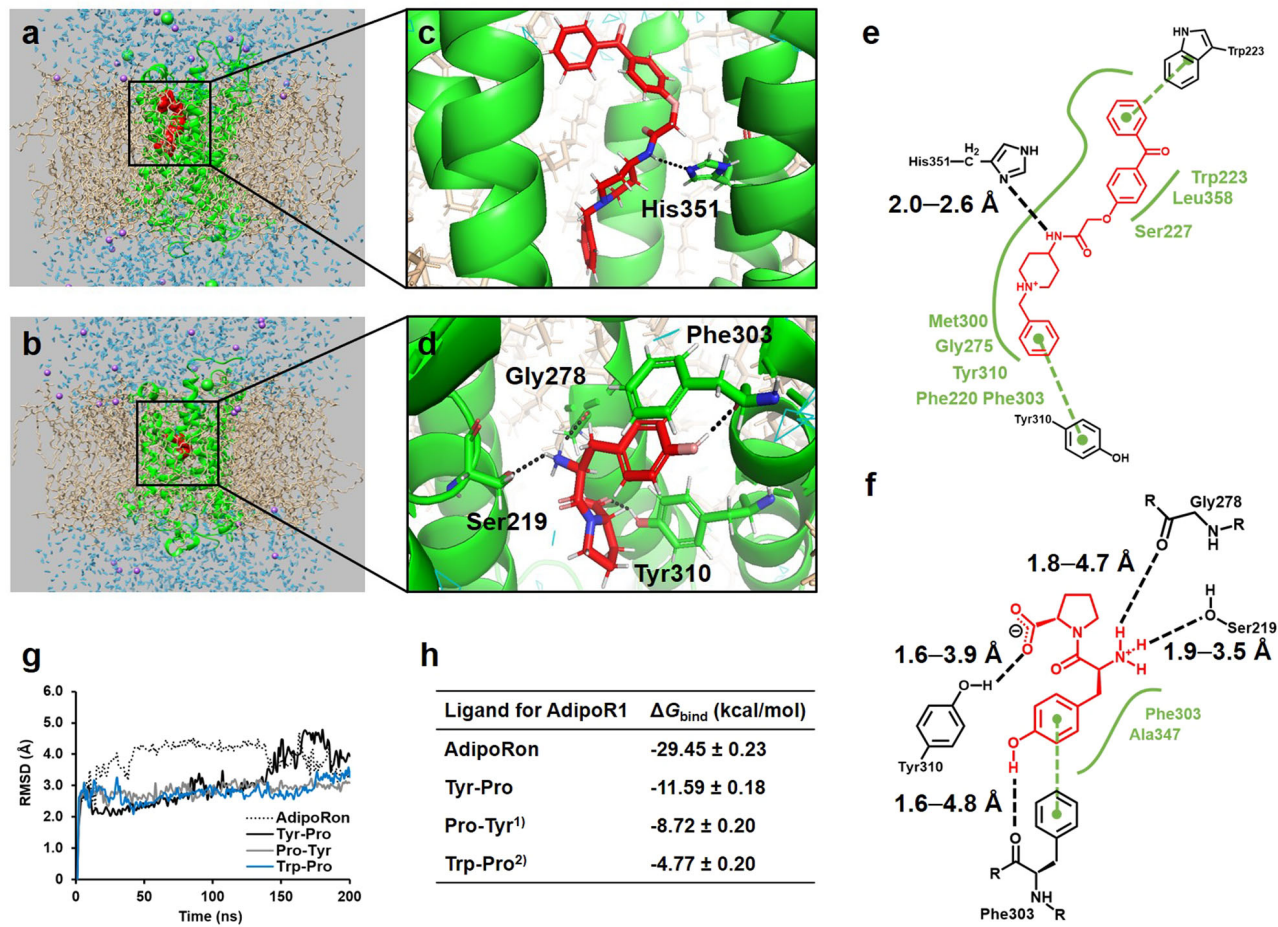
In silico analyses at site 1. MD simulation analyses of the CHARMM-GUI-guided (**a**) AdipoRon–AdipoR1–POPC complex and (**b**) the Tyr-Pro–AdipoR1–POPC complex at site 1 of AdipoR1, visualized using UCSF Chimera ver. 1.14. The corresponding colors are as follows: Na^+^, green sphere, Cl^−^, purple sphere; POPC, yellow; water molecule, cyan. The zoomed view snapshot at 200 ns using PyMOL displays the binding conformations of (**c**) AdipoRon and (**d**) Tyr-Pro complexes. The corresponding colors are as follows: C atom, red; H atom, white; N atom, blue; O atom, pink. Intermolecular interactions of (**e**) AdipoRon and (**f**) Tyr-Pro complexes at 200 ns were visualized using Protein*Plus*. Binding conformations are as follows: hydrogen bond, black dashed line; hydrophobic interaction, green straight line; π–π electron interaction, green dashed line. **g** A protein backbone root-mean-square deviation (RMSD) value from the initial structure of the ligand–AdipoR1–POPC complex was computed for 200 ns. **h** The average binding free energy scores for 180–200 ns, Δ*G*_bind_ (kcal/mol) of AdipoRon–AdipoR1, Tyr-Pro–AdipoR1, Pro-Tyr–AdipoR1, and Trp-Pro–AdipoR1 complexes in POPC. ^1)^MD simulation analyses for Pro-Tyr at site 1 can be seen in Fig. S7 and Movie S3. ^2)^MD simulation analyses for Trp-Pro at site 1 can be seen in Fig. S7 and Movie S4.

**Fig. 6:**
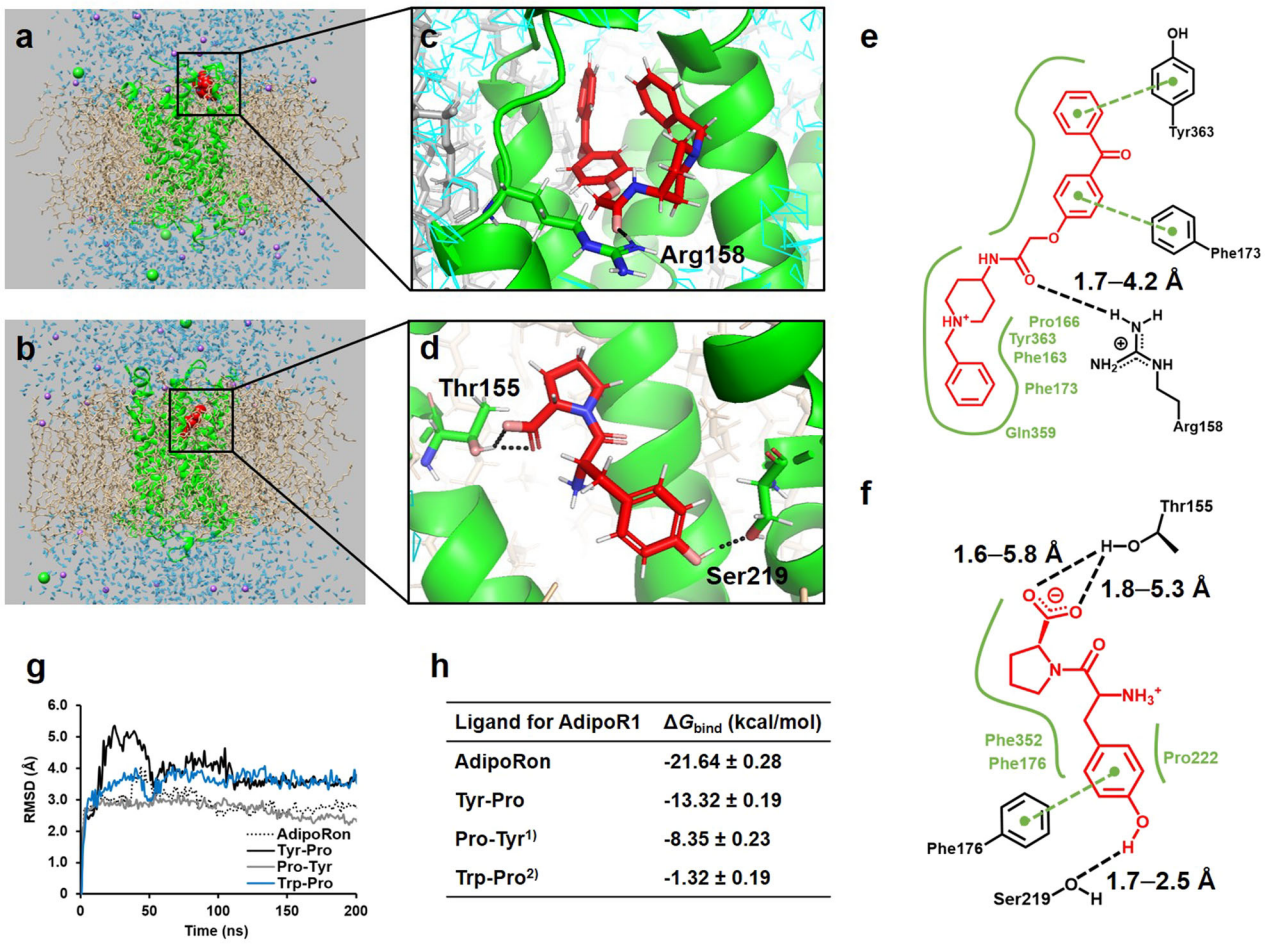
In silico analyses at site 2. MD simulation analyses of CHARMM-GUI-guided (**a**) AdipoRon–AdipoR1–POPC complex and (**b**) Tyr-Pro–AdipoR1–POPC complex at site 2 of AdipoR1, visualized using UCSF Chimera ver. 1.14. The zoomed view snapshot at 200 ns using PyMOL displays the binding conformations of (**c**) AdipoRon and (**d**) Tyr-Pro complexes. The corresponding colors are as follows: C atom, red; H atom, white; N atom, blue; O atom, pink. Intermolecular interactions of (**e**) AdipoRon and (**f**) Tyr-Pro complexes at 200 ns were visualized using Protein*Plus*. Binding conformations are as follows: hydrogen bond, black dashed line; hydrophobic interaction, green straight line; π–π electron interaction, green dashed line. **g** A protein backbone root-mean-square deviation (RMSD) value from the initial structure of the ligand–AdipoR1–POPC complex was computed for 200 ns. **h** The average binding free energy scores for 180–200 ns, Δ*G*_bind_ (kcal/mol) of AdipoRon–AdipoR1, Tyr-Pro–AdipoR1, Pro-Tyr–AdipoR1, and Trp-Pro–AdipoR1 complexes in POPC. ^1)^The MD simulation analyses for Pro-Tyr at site 2 can be seen in Fig. S7 and Movie S7. ^2)^The MD simulation analyses for Trp-Pro at site 2 can be seen in Fig. S7 and Movie S8.

To demonstrate the structural requirements of Tyr-Pro for the AdipoR1 agonistic effect (Fig. 3b), we performed in silico MD simulation analysis of Tyr-Pro with the seven-transmembrane receptor, AdipoR1, anchored in a CHARMM-GUI-aided 1-palmitoyl-2-oleoyl-phosphatidylcholine (POPC) phospholipid membrane (Figs. 5a–f and 6a–f). MD-based docking simulations of the dipeptide–AdipoR1 complex were performed at two potential binding sites (Fig. 5 for site 1, Fig. 6 for site 2) in AdipoR1, which can interact with adiponectin^17^. As depicted in Figs. 5g and 6g, virtual complexes of AdipoRon, Tyr-Pro, Pro-Tyr, and Trp-Pro with the AdipoR1 protein anchored in the POPC membrane provided constant protein backbone root-mean-square deviation (RMSD) values of 2.0–5.0 Å for MD simulations at sites 1 and 2, respectively. Considering the results from previous studies that the protein RMSD value in MD simulation was stable at 2.0–9.0 Å^23^, the virtual systems developed in this study between targets and the AdipoR1 receptor were stably simulated during the MD docking analyses (Movies S1–8). The virtually complexed forms also revealed that the AdipoR1 agonistic Tyr-Pro dipeptide was stabilized at the inner position of each pocket in AdipoR1 (Figs. 5b and 6b, respectively), compared with the position of AdipoRon (Figs. 5a and 6a); at site 1, AdipoRon formed a hydrogen bond with His^351^, hydrophobic interactions with Phe^220^, Trp^223^, Ser^227^, Gly^275^, Met^300^, Phe^303^, Tyr^310^, and Leu^358^, and π–π electron interactions with Tyr^310^ and Trp^223^ (Fig. 5e), while Tyr-Pro formed hydrogen bonds with Ser^219^, Gly^278^, Phe^303^, and Tyr^310^, hydrophobic interactions with Phe^303^ and Ala^347^, and π–π electron interactions with Phe^303^ (Fig. 5f). At site 2, it was simulated that AdipoRon formed a hydrogen bond with Arg^158^, hydrophobic interactions with Phe^163^, Pro^166^, Phe^173^, Gln^359^, and Tyr^363^, and π–π electron interactions with Phe^173^ and Tyr^363^ (Fig. 6e), while Tyr-Pro formed hydrogen bonds with Thr^155^ and Ser^219^, hydrophobic bonds with Phe^176^, Pro^222^, and Phe^352^, and π–π electron interactions with Phe^176^ (Fig. 6f). The magnitude of intermolecular interactions between the four targets and AdipoR1 was evaluated by the binding free energy (Δ*G*_bind_) of the complex during the last 20 ns RMSD simulation (180–200 ns, 200 frames), using the molecular mechanics Poisson–Boltzmann surface area (MM-PBSA) method. The apparent Δ*G*_bind_ of the AdipoRon–AdipoR1 complex at site 1 (−29.45 kcal/mol, Fig. 5h) and site 2 (−21.64 kcal/mol, Fig. 6h) was in good agreement with a potent AdipoR1 agonistic effect (Fig. 3b), demonstrating that AdipoRon, a molecularly designed AdipoR1 agonist^7^, preferably binds to the two pockets of the seven-transmembrane AdipoR1 receptor. Less Δ*G*_bind_ of Trp-Pro (Figs. 5h and 6h), exerting no promoting effect on glucose uptake (Fig. 1c), also validated the current MD simulation for the virtual ligand–AdipoR1 complex. The Δ*G*_bind_ of Tyr-Pro–AdipoR1 complex at site 1 (−11.59 kcal/mol) and site 2 (−13.32 kcal/mol) demonstrated the favorable agonistic action of Tyr-Pro towards the two pockets of AdipoR1 (Fig. 3b), similar to AdipoRon. The unexpected result that Pro–Tyr, with no in vitro bioactivity in L6 myotubes (Fig. 1b), had a compatible Δ*G*_bind_ at site 1 (−8.72 kcal/mol) and site 2 (−8.35 kcal/mol) with those of Tyr-Pro obtained from in silico analysis. The conflicting results between in vitro and in silico experiments might be explained by the degradation (or enzymatic hydrolysis) of Pro–Tyr during the 1 h-glucose uptake experiments (Fig. S6). Moreover, provided that no degradation of Pro-Tyr occurred during the transport experiments, Pro-Tyr must be a potent AdipoR1 agonist, as well as Tyr-Pro (Movies S3 and S7, Fig. S7). Taken together, the in silico MD simulation analyses of Tyr-Pro (Figs. 5 and 6) and Pro-Tyr (Fig. S7), and Trp-Pro (Fig. S7) at the pockets of AdipoR1 revealed that both intermolecular interactions by π–π electron interactions and hydrogen bonds between Tyr and the pockets would elicit the AdipoR1 agonistic effect of Pro-dipeptides.

## Discussion

This study evidenced that a dipeptide, Tyr-Pro, can promote glucose uptake in L6 myotubes by acting as an adiponectin-like AdipoR1 agonist to simulate the AMPK/Glut4 translocation cascade. The great advantage of the adiponectin-like therapeutic strategy for NIDDM treatment is that it does not consider impaired insulin sensitivity or reduced adiponectin secretion. Okada-Iwabu et al. successfully developed the adiponectin-like therapeutic agent, AdipoRon, and demonstrated in vivo anti-diabetic effects in *db*/*db* mice, showing reduced adiponectin secretion by a high-fat diet^7^. The orallyactive anti-diabetic effect of AdipoRon resulted from its small molecular size (MW, 428.5) and high affinity for the target AdipoR1 receptor by its characteristic 1-benzyl 4-substituted 6-membered cyclic amine moiety and aromatic rings linked with amide bonds (Fig. 1a). Referring to the structural requirements of AdipoRon for the AdipoR1 agonist, we successfully discovered a dipeptide agonist, Tyr-Pro, from 15 synthetic dipeptides bearing a cyclic amine moiety and aromatic ring linked with peptide bonds in the structure. Considering that the glucose incorporation of 0.1 µM AdipoRon (162 ± 6%) was comparable with 1 µM Tyr–Pro (166 ± 7%) (Fig. 4a), the glucose-promoting potential of the Tyr-Pro dipeptide seems to be approximately 1/10-lower than that of AdipoRon. Among the reported natural compounds with glucose-promoting effects, Tyr-Pro present in soybean hydrolysate at 0.47 mg/g-hydrolysate^24^ may be categorized as a potent promotor for glucose uptake, since other natural compounds, such as 6-*O*-caffeoylsophorose (179 ± 18%, 100 µM, 1 h)^25^, theasinensins (ca. 200%, 50 µM, 1 h)^4^, naringenin (193 ± 24%, 75 µM, 2 h)^26^, and rosmarinic acid (186 ± 4%, 5 µM, 4 h)^27^, showed weaker glucose uptake potency in L6 myotubes than that of Tyr-Pro (174 ± 23%, 1 µM, 1 h) (Fig. 2b).

The complete disappearance of the promotion of glucose uptake by Tyr-Pro in AdipoR1-knockdown L6 myotubes (Fig. 3b) indicates the preferential agonistic binding to the AdipoR1 receptor, which is different from the dipeptide (Trp-His)-induced glucose uptake via PHT1-mediated upregulation of intracellular signaling towards AMPK/Glut4 translocation^5^. To date, Kim et al. ^17^ applied a docking simulation of ligands with AdipoR1 crystal structures to clarify the configuration of adiponectin binding sites and successfully simulated the involvement of two pores (sites 1 and 2) of seven-transmembrane AdipoR1 in the docking of adiponectin and designed 15-mer oligopeptides. In the present CHARMM-GUI-aided ligand–AdipoR1 complex in POPC membranes under virtual physiological conditions (Movies 1–8), we confirmed that Tyr-Pro and AdipoRon could preferably and non-selectively bind to the two pores of AdipoR1 (Figs. 5 and 6), similar to adiponectin^17^. The AutoDock-aided simulation of the Tyr-Pro–AdipoR1 complex in a virtual grid-box region provided further useful information that Tyr-Pro was stably anchored at the inner pockets of each site, compared to the position of AdipoRon (Figs. 5 and 6, and Movies 1, 2, 5, and 6). Low Δ*G*_bind_ of <−8 kcal/mol for Tyr-Pro as well as Pro-Tyr, estimated by the MM-PBSA method, may be explained by the key-player, Tyr, in their sequences for binding to AdipoR1 pores, in which the presence of a hydroxyl group and an aromatic ring of Tyr may greatly influence the highly stability of the Pro-dipeptides at each site of AdipoR1 by an intermolecular hydrogen bond and π–π electron interactions (Figs. 5 and 6). The high Δ*G*_bind_ of >−5 kcal/mol for Trp-Pro may be due to the lack of the above two intermolecular interactions between Trp and the receptor at the two pores (Fig. S7).

It was apparent that the conflicting results between in silico activity (Figs. 5 and 6) and in vitro inactivity of Pro-Tyr (Fig. 1b) were caused by its poor stability or enzymatic degradation in L6 cell experiments (Fig. S6). In our peptide absorption studies^18–22^, we have revealed that intact absorbable peptides in the circulating bloodstream, such as Trp-His^20^ and Val-Tyr^28^, possessed high protease resistance at the apical side of the brush border beyond the affinity with PepT1^20,29,30^. This study examined AdipoR1-agonistic dipeptides rather than longer oligopeptides using in vitro and in silico analyses because of their favorable absorption^22^. The bioavailability and anti-diabetic effect of Tyr-Pro should be investigated not only in muscles but also in other AdipoR1-expressed organs, such heart^31^, lung^32^, and kidney^33^, if it is orally active, and oral administration experiments using SDT rats are now underway. Oligopeptides of more than a 3-mer can be a targeting candidate, since oligopeptides, such as the ubiquitin ligase inhibitory pentapeptide, DGYMP^34^, egg white-derived antihypertensive tripeptide, IRW^35^, and bovine collagen-derived angiotensin I-converting enzyme-inhibitory peptides, VGPV and GPRGF^36^, are reportedly absorbed in their intact forms. Thus, further experiments on possible AdipoR1-agonistic peptides were performed using synthetic tripeptides based on Tyr-Pro as a skeleton.

In conclusion, we have demonstrated a dipeptide, Tyr-Pro, exhibiting AdipoR agonistic effects by binding to two pores of AdipoR1 by in vitro L6 cell and in silico MD simulation experiments. It can be simulated that the Tyr residue of Pro-dipeptides may play a crucial role in the stability at the two pores of AdipoR1 by its intermolecular hydrogen bonds and π–π electron interactions. The evidence from this study can greatly contribute to the prevention of NIDDM with obesity-related glucose tolerance by adiponectin-like “orally active” peptide agonists.

## Methods

### Materials

Dipeptides (Tyr-Pro, Pro-Tyr, Tyr-His, His-Tyr, Tyr-Trp, Trp-Tyr, Phe-Pro, Pro-Phe, Phe-His, His-Phe, Phe-Trp, Trp-Phe, Ile-Pro, Trp-Pro, and His-Pro) were purchased from Kokusan Chemical (Tokyo, Japan). AdipoRon was obtained from Cayman Chemical (Ann Arbor, MI, USA). Insulin and dimethyl sulfoxide (DMSO, 99.9%) were purchased from Sigma-Aldrich (St. Louis, MO, USA). Dulbecco’s modified Eagle’s medium (DMEM), fetal bovine serum (FBS), and horse serum (HS) were obtained from Gibco (Grand Island, NY, USA). Serum-free α-MEM was purchased from Wako Pure Chemicals (Osaka, Japan). 2-(*N*-(7-nitrobenz-2-oxa-1,3-diazol-4-yl) amino)-2-deoxyglucose (2-NBDG) was purchased from Invitrogen (Carlsbad, CA, USA). Compound C was purchased from Merck Millipore (Darmstadt, Germany). Other chemicals were of analytical grade and used without further purification.

### Cell culture

Rat skeletal muscle L6 myoblasts (JCRB9081), were obtained from the Japanese Collection of Research Bioresources (JCRB Cell Bank, Osaka, Japan). L6 myoblasts were grown in DMEM containing 10% FBS (v/v) at 37 °C in a humidified atmosphere with 5% CO_2_. For differentiation into myotubes, L6 cells were transferred to DMEM containing 2% HS (v/v). The medium was refreshed every day to obtain mature L6 myotubes for at least 5 days. Before each experiment, the medium was replaced with serum-free α-MEM containing 0.2% bovine serum albumin (BSA, w/v) for 18 h.

### Cell viability

Cell viability was assessed using a Cell Counting kit-8 (CCK-8, Dojindo Molecular Technologies, Kumamoto, Japan). Briefly, L6 cells (1,500 cells/well in a 96-well microplate) were exposed to different Tyr-Pro concentrations (0.1–10 µM) for 2 h and then treated with 10 µL of CCK-8 solution for an additional 3 h. Absorbance was measured at 450 nm using a Wallac 1420 microplate reader (Perkin-Elmer, Waltham, MA, USA).

### Glucose uptake assay

Glucose uptake was assessed using a 2-NBDG fluorescence assay according to a previously described method^4^, with minor modifications. L6 myoblasts were seeded at 1,500 cells/well in a 96-well microplate and allowed to 80% confluence (48 h). After differentiation, the serum-deprived cells were rinsed twice with Krebs–Ringer’s phosphate HEPES (KRPH) buffer: 118 mM NaCl, 5 mM KCl, 1.2 mM KH_2_PO_4_, 1.3 mM CaCl_2_, 1.2 mM MgSO_4_, and 30 mM HEPES (pH 7.4). The cells were then followed by incubation with 100 µL of the dipeptide (0.1–10 µM, 0.5–2 h) in KRPH buffer containing 0.1% DMSO (v/v). An AMPK inhibition study was performed by adding 20 µM compound C to the dipeptide solution. AdipoRon (0.1 µM) and insulin (0.1 µM) were used to validate the 2-NBDG uptake experiments in L6 myotubes (0.5 h, positive controls). Next, 100 µL of 100 µM 2-NBDG solution (KRPH buffer) was added to each well and incubated for 1 h at 37 °C. At the end of the treatment, the cells were washed three times with ice-cold KRPH buffer. The fluorescence intensity of 2-NBDG was detected at excitation and emission wavelengths of 465 and 540 nm, respectively, using a Flex Station II scanning fluorometer (Molecular Devices, Sunnyvale, CA, USA). The level of 2-NBDG uptake was represented as a percentage of the relative fluorescence intensity of the control.

### AdipoR1 siRNA knockdown

Transfection of L6 cells with either AdipoR1 siRNA (sc-156024, Lot: B2216; Santa Cruz, CA, USA) or negative control siRNA (sc-37007, Lot: 11418; Santa Cruz, CA, USA) was performed according to the manufacturer’s protocol with slight modifications. Briefly, L6 cells were seeded at 1,500 cells/well in a 96-well microplate (for glucose uptake analysis) or 1 × 10^5^ cells/well in a 6-well microplate (for immunodetection analysis) and then transfected with siRNA at a final concentration of 20 nM, using a Hiperfect transfection reagent (Qiagen, Hilden, Germany). After 6 h, the medium was replaced with fresh differentiation medium. The cells were incubated for an additional 5 days and then re-transfected using the same method. Subsequently, the transfected cells were used for either glucose uptake or immunodetection analyses.

For detection of AdipoR1, the transfected cells were washed with ice-cold PBS, lysed using 2 × radioimmunoprecipitation assay (RIPA) buffer (100 mM Tris-HCl, pH 8.0, 300 mM NaCl, 2% (v/v) Nonidet P-40 (NP-40), 1% (w/v) sodium deoxycholate, and 0.2% (w/v) sodium dodecyl sulfate) with a protease inhibitor cocktail (Nacalai Tesque, Kyoto, Japan) and phosphatase inhibitor cocktail tablets (PhosSTOP, Roche, Basel, Switzerland), and homogenized with a polytron homogenizer (KINEMATICA AG, Luzern, Switzerland) at 20,000 rpm for 30 s at 4 °C. The homogenate was then centrifuged (20,000 × *g*, 20 min, 4 °C) to obtain the supernatant as the whole cell lysate.

### AMPK phosphorylation

L6 myoblasts (1 × 10^5^ cells/well in a 6-well microplate) were differentiated into myotubes and then treated with 1 µM Tyr-Pro for 1 h. A 0.5 h-incubation with 0.1 µM AdipoRon was considered as a positive control. After being treated with the samples, the myotubes were washed with ice-cold PBS, lysed with 2 × RIPA buffer with protease and phosphatase inhibitors, and then centrifuged (20,000×*g*, 20 min, 4 °C) to obtain the supernatant as the whole cell lysate.

### Glut4 translocation assays

L6 myoblasts (4 × 10^5^ cells/well in a 6-well microplate) were differentiated into myotubes and then treated with either 1 µM Tyr-Pro for 1 h or 0.1 µM AdipoRon for 0.5 h, in the presence or absence of 20 µM compound C. For detecting the level of Glut4 translocation in the cells, the plasma membrane (PM) fraction was extracted by the previous method^37^ with several modifications. After rinsing two times with ice-cold PBS, the samples treated with L6 myotubes were harvested in ice-cold lysis buffer (50 mM Tris-HCl, 0.5 mM dithiothreitol, pH 8.0) containing 0.1% (v/v) NP-40 and protease and phosphatase inhibitor cocktails and homogenized. The homogenate was then centrifuged (1,000×*g*, 10 min, 4 °C) to obtain a pellet. The pellet was suspended in lysis buffer with the same inhibitors, kept on ice for 10 min, and then centrifuged (1,000×*g*, 10 min, 4 °C). The resulting pellet was resuspended in lysis buffer containing 1% (v/v) NP-40 and the same inhibitors on ice for 1 h with occasional mixing. After centrifugation (20,000 × *g*, 20 min, 4 °C), the supernatant was collected as the PM fraction. To extract the whole cell lysate, part of the homogenate prepared as described above was mixed with an equal volume of 2 × RIPA buffer with the same inhibitors and kept on ice for 1 h with occasional mixing. Then, it was centrifuged (20,000×*g*, 20 min, 4 °C), and the supernatant was used as the whole cell lysate.

### The Wes analysis

Target protein levels were measured using a capillary electrophoretic-based immunoassay (the Wes instrument; ProteinSimple Co., San Jose, CA, USA), according to the manufacturer’s protocol with slight modifications. Briefly, samples were combined with 0.1 × sample diluent buffer and 5× fluorescent master mix denaturing buffer to acquire 0.5 µg/µL loading concentration (except for Glut4 detection: 0.8 µg/µL of cell lysate, 2.0 µg/µL of PM). Then, the samples were denatured to avoid protein aggregation by 5 min at 95 °C (except for Glut4 detection: 20 min, 37 °C). The primary antibodies used in this study were as follows: AMPK (1:100 dilution, anti-rabbit, 07-350, Lot: 2922422; Merck Millipore, Darmstadt, Germany), p-AMPK (1:100 dilution, anti-rabbit, 07-681, Lot: 2901522; Merck Millipore, Darmstadt, Germany), AdipoR1 (1:50 dilution, anti-rabbit, ab126611, Lot: GR277137-17; Abcam, Cambridge, UK), and Glut4 (1:10 dilution, anti-mouse, 2213S, Lot: 7; Cell Signaling Technology, Danvers, MA, USA). The Wes measurement was performed using a 12–230 kDa separation module (8 × 25 mm capillary cartridge, ProteinSimple Co.). The Wes reagents, including a biotinylated ladder (marker) and primary antibodies, were dispensed in a microplate and subjected to Wes automated capillary electrophoresis, followed by immune detection using a horseradish peroxidase-conjugated secondary antibody and a chemiluminescent substrate. For AdipoR1 and Glut4 detection, a total protein assay was used to normalize the target protein signal. At the end of the run, the chemiluminescent signal was displayed as a virtual blot-like image and electropherogram based on the molecular weight using a Compass software (ProteinSimple Co.). The virtual blot-like images derive from the same experiment and were processed in parallel.

### Preparation of ligands

The initial structure of AdipoRon (CID 16307093) was acquired from PubChem (https://pubchem.ncbi.nlm.nih.gov/), and its geometry was optimized using Gaussian ver. 16A. 03 at the B3LYP/6–31G(d) level. The antechamber implemented in AMBER18 was used to generate the restrained ESP charges of the ligands. Tyr-Pro was built using the UCSF Chimera ver. 1.14, and it was also mutated to create other ligands (Pro-Tyr and Trp-Pro) using a CHARMM-GUI-mutation (http://www.charmm-gui.org/)^38^.

### Preparation of the AdipoR1 protein

Although rat-derived L6 myotubes were used for in vitro analysis, the crystal structure of human AdipoR1 protein (ID 3WXV) from the RCSB Protein Data Bank (https://www.rcsb.org/) was utilized as a template for ligand docking studies. The sequence of human- and rat-derived AdipoR1 is only different at the 373th amino acid, which does not affect the docking analysis (Fig. S4). The template structure of AdipoR1 was modified to create an appropriate protein structure for docking: (1) unnecessary parts (nanobodies) in the template were removed from the initial structure using a PyMOL ver. 2.3.4. (2) To obtain a complete structure of AdipoR1, missing amino acid residues in the template, including Pro^159^, Asn^160^, Gly^298^, and Gln^299^, were reconstructed using a MODELLER ver. 9.23. The input alignment for modeling was obtained using a ClustalW ver. 2.1 based on the human AdipoR1 domain sequence retrieved from the UniProt Knowledgebase (UniProtKB Q96A54, https://www.uniprot.org/), and (3) a missing zinc ion was inserted into the MODELLER-reconstructed seven-transmembrane AdipoR1 protein (Fig. S5).

### Molecular docking and MD simulation

Molecular docking of the ligand in the AdipoR1 protein was performed using an AutoDock Tools ver. 1.5.6. Ligand-binding sites in AdipoR1 were determined according to the information of the two reported pores (sites 1 and 2) of AdipoR1 for the presumed binding of adiponectin^17^. The grid-box parameters for creating the ligand-binding region at each pore are listed in Table S1.

To set a MD simulation of a ligand with a seven-transmembrane AdipoR1 protein anchored into a virtual POPC membrane, the lipid bilayer configuration of the POPC membrane was generated by a CHARMM-GUI (http://www.charmm-gui.org/)^38^. The POPC lipid system was primarily solvated using a transferable intermolecular potential with three points (TIP3P) for water molecules^39^, and then 0.15 M NaCl was added to mimic the physiological condition. The AMBER ff14SB^40^, lipid14^41^, and the general AMBER force field 2^42^ were applied to dipeptides and AdipoR1, POPC, and AdipoRon, respectively. MD simulations of the virtual system were conducted using the AMBER. Minimization and equilibration treatments of ligand-AdipoR1 in POPC membranes were performed using a CHARMM-GUI protocol (310 K, 1 bar)^40^. The simulations with the NPT ensemble were performed at this temperature until the simulation time reached 200 ns. The protein backbone RMSD value of the complex in POPC was used to evaluate the stability of the virtual system.

### Binding free energy (Δ*G*_bind_) of the ligand with AdipoR1

The average binding free energy of solvation (Δ*G*_bind_) between the ligand and AdipoR1 in the POPC system was estimated using the molecular mechanics Poisson–Boltzmann surface area (MM-PBSA) method^43^.

1 $${\Delta}G_{{\mathrm {bind}}} = G_{{\mathrm {complex}}} - \left( {G_{{\mathrm {ligand}}} + G_{{\mathrm {protein}}}} \right)$$

where the *G*_complex_ is the total free energy of the ligand–AdipoR1–POPC complex in the water system, and *G*_ligand_ and *G*_protein_ are the total free energies of the ligand and AdipoR1 alone in the water system, respectively.

### Intermolecular interactions

Intermolecular interactions between ligands and AdipoR1 pores were obtained, and hydrogen bonds, hydrophobic, and π–π electron interactions in the complex were visualized using a Proteins*Plus* server (http://proteins.plus/). The hydrogen bond distances were calculated during 180–200 ns simulation using a UCSF Chimera.

### LC–TOF/MS analysis of dipeptides in the L6 cell medium

The stability of dipeptides (1 µM Tyr-Pro and 1 µM Pro-Tyr) was evaluated in the L6 cell medium using liquid chromatography-time-of-flight/mass spectrometry (LC–TOF/MS) in media obtained before and after 1 h-2-NBDG uptake experiments. The media were passed through a Merck Millipore 0.2 µm-polytetrafluoroethylene membrane filter, and an aliquot (20 µL) of the filtrate was injected into the LC–TOF/MS. The LC system was composed of an Agilent 1200 series HPLC system (Agilent Technologies, Waldbronn, Germany), and LC separation was conducted on a Cosmosil 5C_18_-MS-II column (2.0 × 150 mm, Nacalai Tesque, Kyoto, Japan) with a linear elution of water containing 0.1% formic acid to methanol containing 0.1% formic acid over 20 min at 0.2 mL/min at 40 °C. TOF/MS analysis was performed using a Bruker micrOTOF-II MS instrument (Bruker Daltonics, Bremen, Germany) in electrospray ionization (ESI)-positive ion mode ([M + H]^+^ for Tyr-Pro and Pro-Tyr: 279.1353*m/z*). ESI-MS conditions were set as follows: drying N_2_ gas, 8.0 L/min; drying temperature, 200 °C; nebulizer pressure, 1.6 bar; capillary voltage, 4500 V; mass range, 100–1000*m/z*. A calibration solution (10 mM sodium formate in 50% acetonitrile) was injected at the beginning of each run, and all spectra were calibrated internally. MS data were analyzed using Bruker Data Analysis ver. 3.2 software.

### Statistical analysis

Results from distinct replicates are expressed as the mean ± standard error of the mean (SEM). Statistical differences within groups were evaluated by one-way analysis of variance (ANOVA), followed by Dunnett’s *t*-test. Statistical differences between the two groups were evaluated using unpaired two-tailed Student’s *t*-test. Statistical significance was set at a *p* < 0.05. All analyses were performed using GraphPad Prism ver. 5.0 (La Jolla, CA, USA).

### Reporting summary

Further information on research design is available in the Nature Research Reporting Summary linked to this article.

## Supplementary information

Supplementary Information

Supplementary Movie 1

Supplementary Movie 2

Supplementary Movie 3

Supplementary Movie 4

Supplementary Movie 5

Supplementary Movie 6

Supplementary Movie 7

Supplementary Movie 8

Reporting Summary

## Data Availability

The data supporting the finding reported herein are available on reasonable request from the corresponding author.
