# Supplementary material for: In vitro and in silico characterization of adiponectin-receptor agonist dipeptides
Source: NPJ Sci Food. 2021 Nov 12;5:29. doi: 10.1038/s41538-021-00114-2 (PMC8589863; doi:10.1038/s41538-021-00114-2)
Supplement: Supplementary file 1 — Supplementary Information [file 41538_2021_114_MOESM1_ESM.pdf]

Supplementary information

In vitro and *in silico* characterization of adiponectin-receptor agonist dipeptides

Yuna Lee[1], Akihiro Nakano[1], Saya Nakamura[1], Kenta Sakai[2], Mitsuru Tanaka[2], Keisuke Sanematsu[2,3,4], Noriatsu Shigemura[2,3], Toshiro Matsui[1,2]*

[1]Department of Bioresources and Biosciences, Faculty of Agriculture, Graduate School of Kyushu University, 744 Motooka, Nishi-ku, Fukuoka 819-0395, Japan

[2]Research and Development Center for Five-Sense Devices, Kyushu University, 744 Motooka, Nishi-ku, Fukuoka 819-0395, Japan

[3]Section of Oral Neuroscience, Graduate School of Dental Science, Kyushu University, 3-1-1 Maidashi, Higashi-ku, Fukuoka 812-8582, Japan

[4]Oral Health/Brain Health/Total Health Research Center, Graduate School of Dental Science, Kyushu University, 3-1-1 Maidashi, Higashi-ku, Fukuoka 812-8582, Japan

Running head: Adiponectin-receptor agonist dipeptides

*Correspondence: Professor Toshiro Matsui, Department of Bioresources and Biosciences, Faculty of Agriculture, Graduate School of Kyushu university, 744 Motooka, Fukuoka 819-0395, Japan

Tel./fax: +81-92-802-4752, E–mail: tmatsui@agr.kyushu-u.ac.jp (T. Matsui)

Supplementary Figures

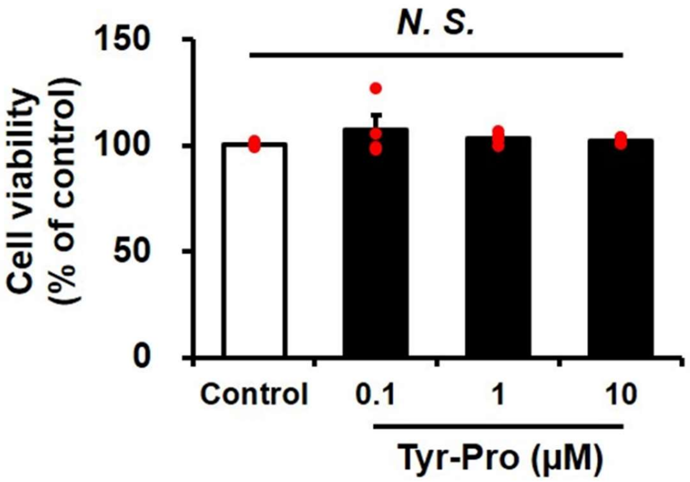

Supplementary Figure 1. The effect of Tyr-Pro on L6 cell viability. The viability of L6 cells following Tyr-Pro treatment was evaluated by a cell counting kit-8 (CCK-8) assay. Results were expressed as the mean ± SEM (n = 4). Statistical differences were evaluated by unpaired two-tailed Student's t-test; *N. S.*, no significant difference at $p > 0.05$.

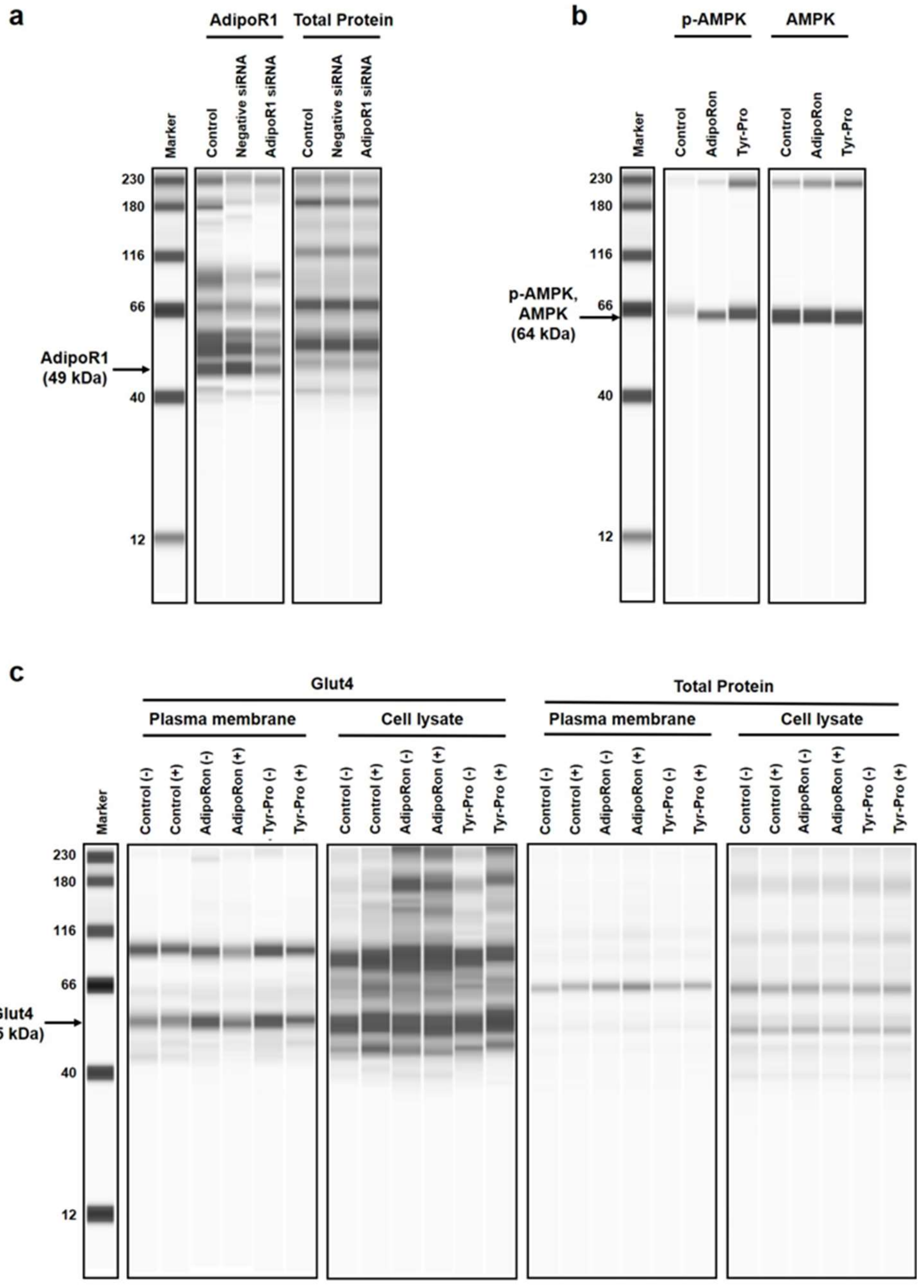

Supplementary Figure 2. Uncropped virtual blot-like images by a Wes analysis. The protein expressions of (a) AdipoR1, (b) p-AMPK and AMPK, and (c) Glut4 were evaluated by a Wes instrument, as described in Methods section. In (c), (-) and (+) indicate the absence and presence of AMPK inhibitor, respectively. The uncropped virtual images used in Figures 3 and 4 are provided.

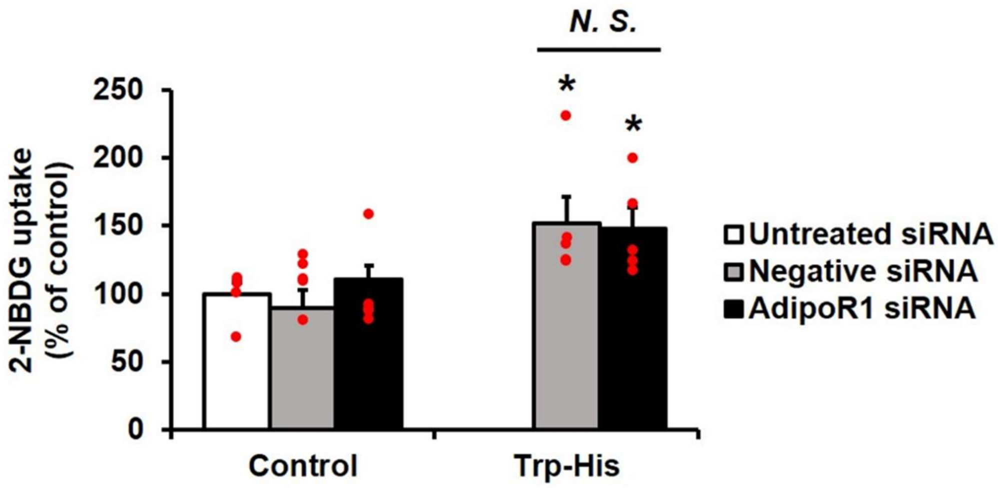

Supplementary Figure 3. The effect of AdipoR1-knockdown in L6 myotubes on the promotion of 2-NBDG uptake by Trp-His. The knocked-down L6 myotubes were incubated with 10 μM Trp-His for 1h for 2-NBDG uptake experiments. Results are expressed as the mean ± SEM (n = 5). Statistical differences were evaluated using Dunnett's t-test. *p < 0.05 vs. control. *N. S.*, no significant difference by unpaired two-tailed Student's t-test between negative and AdipoR1 siRNA groups at p > 0.05.

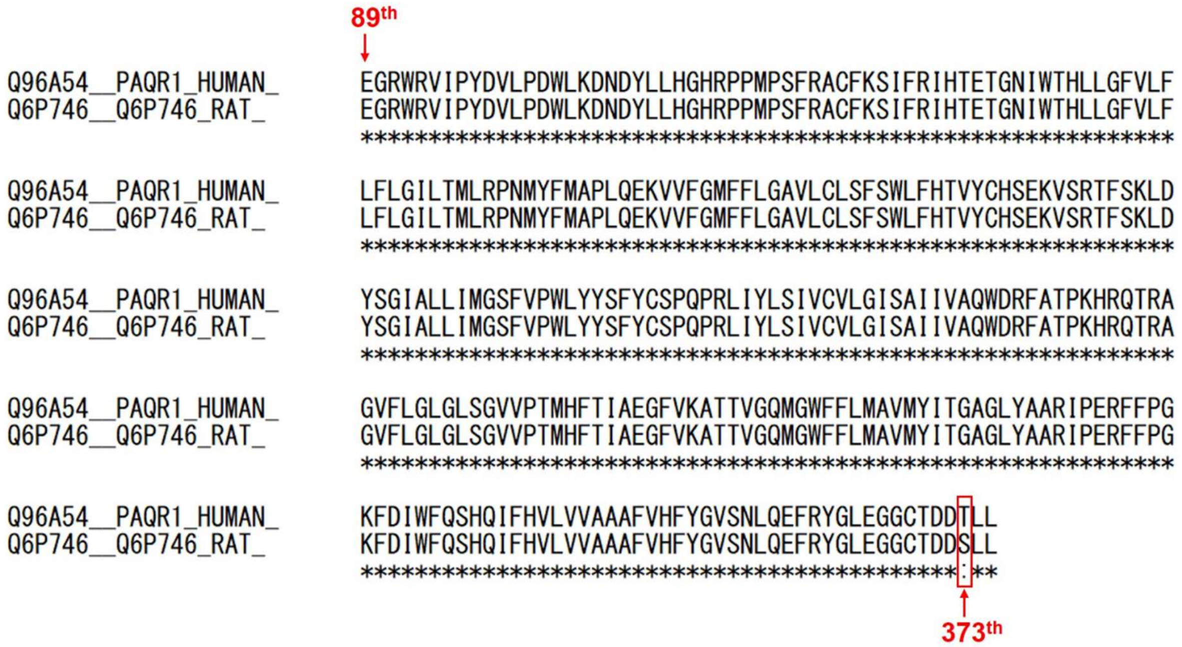

Supplementary Figure 4. Sequence alignments of the human AdipoR1 (UniProtKB Q96A54) and rat AdipoR1 (UniProtKB Q6P746) using ClustalW ver. 2.1. While the same amino acid residues were highlighted with "*", the different residues were marked using a ":" symbol (red square).

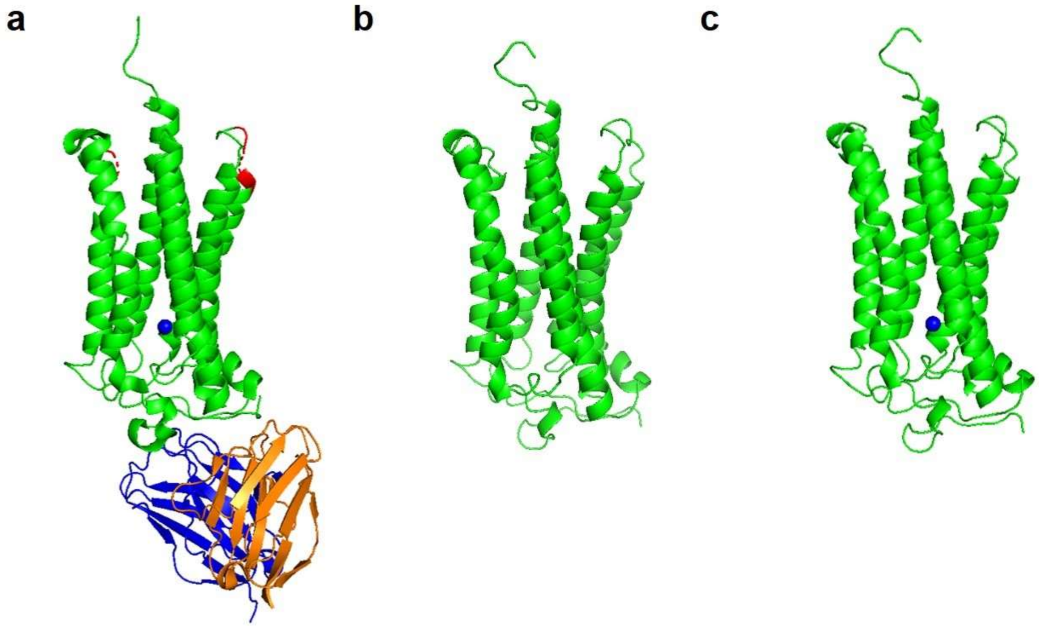

Supplementary Figure 5. The three-dimensional structure of AdipoR1 visualized using Chimera ver. 1.14. (a) The initial structure of AdipoR1 (PDB ID 3WXV) including missing amino acid residues (red regions). (b) AdipoR1 modeling for repairing the missing amino acid residues. **(c)** Reconstruction of the AdipoR1 model by the addition of a missing zinc ion (blue sphere).

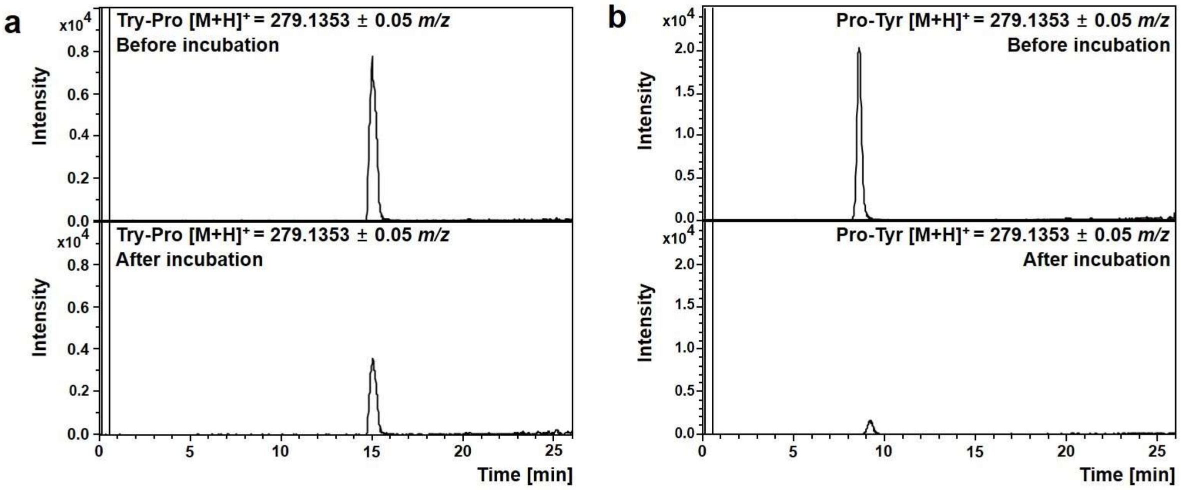

Supplementary Figure 6. LC-TOF/MS chromatograms in the EIC mode of dipeptides (Tyr-Pro and Pro-Tyr) obtained from medium solution before and after 2-NBDG uptake experiments for 1 h; (a) Tyr-Pro (1 μM), 279.1353 *m/z*, (b) Pro-Tyr (1 μM), 279.1353 *m/z*.

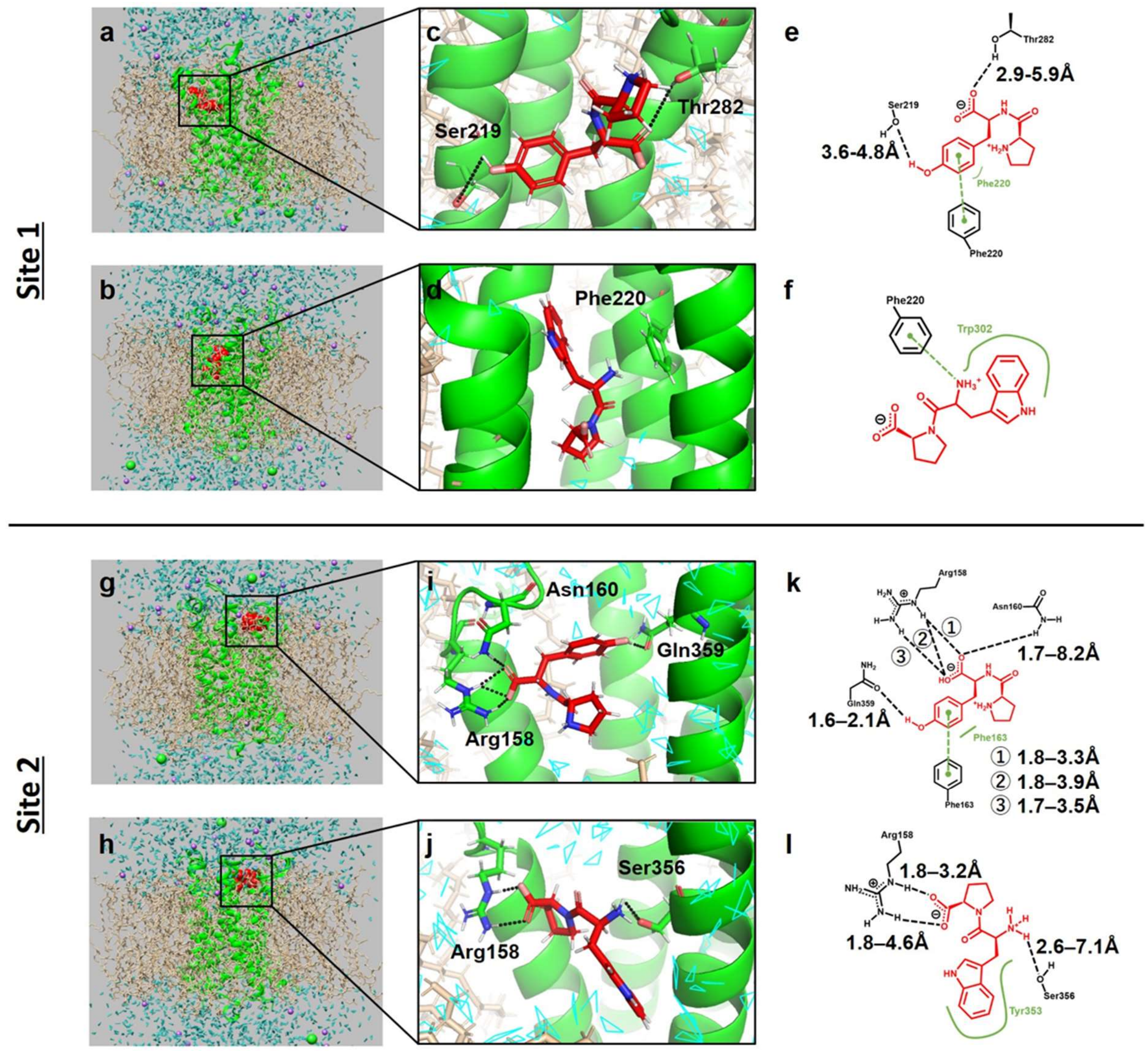

Supplementary Figure 7. *In silico* analyses at site 1 and 2. MD simulation analyses of CHARMM-GUI-guided (a) Pro-Tyr-AdipoR1-POPC complex and (b) Trp-Pro-AdipoR1-POPC complex at site 1 of AdipoR1, visualized using UCSF Chimera ver. 1.14. The corresponding colors are as follows: Na^+, green sphere, Cl^-, purple sphere; POPC, yellow; water molecule, cyan. The zoomed view snapshot using PyMOL displays the binding conformations of (c) Pro-Tyr (T = 196 ns) and (d) Trp-Pro (T = 193 ns) complexes. The corresponding colors are as follows: C atom, red; H atom, white; N atom, blue; O atom, pink. Intermolecular interactions of (e) Pro-Tyr and (f) Trp-Pro complexes at 200 ns were visualized

using Protein*Plus*. Binding conformations are as follows: hydrogen bond, black dashed line; hydrophobic interaction, green straight line; π–π electron interaction, green dashed line.

MD simulation analyses of CHARMM-GUI-guided (g) Pro-Tyr-AdipoR1-POPC complex and (h) Trp-Pro-AdipoR1-POPC complex at site 2 of AdipoR1, visualized using UCSF Chimera ver. 1.14. The corresponding colors are as follows: Na^{+}, green sphere, Cl^{-}, purple sphere; POPC, yellow; water molecule, cyan. The zoomed view snapshot at 200 ns using PyMOL displays the binding conformations of (i) Pro-Tyr and (j) Trp-Pro complexes. The corresponding colors are as follows: C atom red; H atom, white; N atom, blue; O atom, pink. Intermolecular interactions of (k) Pro-Tyr and (l) Trp-Pro complexes at 200 ns were visualized using Protein*Plus*. The binding conformations are as follows: hydrogen bond, black dashed line; hydrophobic interaction, green straight line; π–π electron interaction, green dashed line.

Supplementary Table

Supplementary Table 1. Grid-box coordinates and size parameters used in AutoDock Tools ver. 1.5.6.

Grid-box parameters		Site 1	Site 2
Center (Å)	*x*	21.654	21.959
	y	64.273	59.570
	z	3.079	-7.978
Size (Å)	*x*	14	10
	y	28	22
	z	8	8

Supplementary Movies

Supplementary Movie 1. An MD simulation movie of the AdipoRon-AdipoR1-POPC complex for 200 ns at site 1.

Supplementary Movie 2. An MD simulation movie of the Tyr-Pro-AdipoR1-POPC complex for 200 ns at site 1.

Supplementary Movie 3. An MD simulation movie of the Pro-Tyr-AdipoR1-POPC complex for 200 ns at site1.

Supplementary Movie 4. An MD simulation movie of the Trp-Pro-AdipoR1-POPC complex for 200 ns at site1.

Supplementary Movie 5. An MD simulation movie of the AdipoRon-AdipoR1-POPC complex for 200 ns at site 2.

Supplementary Movie 6. An MD simulation movie of the Tyr-Pro-AdipoR1-POPC complex for 200 ns at site 2.

Supplementary Movie 7. An MD simulation movie of the Pro-Tyr-AdipoR1-POPC complex for 200 ns at site 2.

Supplementary Movie 8. An MD simulation movie of the Trp-Pro-AdipoR1-POPC complex for 200 ns at site 2.
