# Supplementary figures and images for: In vitro and in silico characterization of adiponectin-receptor agonist dipeptides
Source: NPJ Sci Food. 2021 Nov 12;5:29. doi: 10.1038/s41538-021-00114-2 (PMC8589863; doi:10.1038/s41538-021-00114-2)

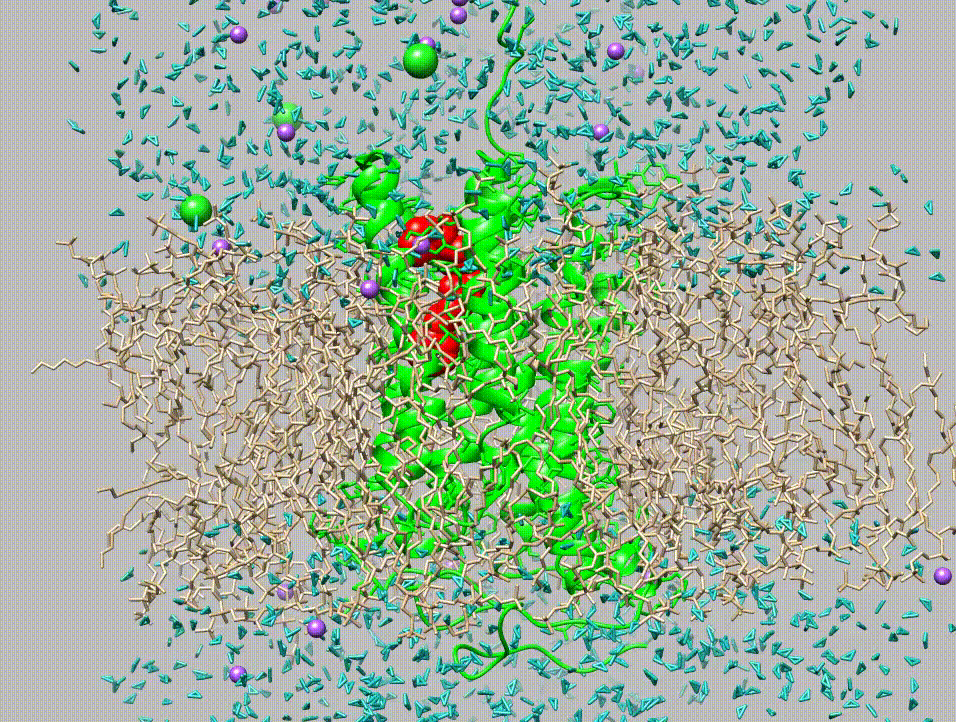

Supplement: Supplementary file 2 — Supplementary Movie 1 [file 41538_2021_114_MOESM2_ESM.gif]

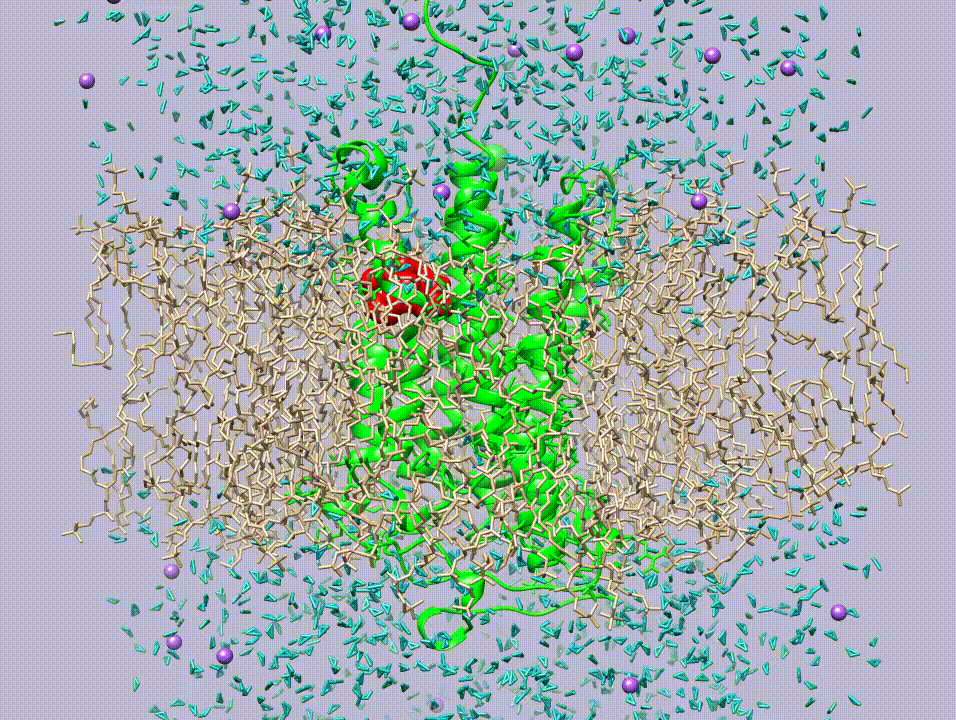

Supplement: Supplementary file 3 — Supplementary Movie 2 [file 41538_2021_114_MOESM3_ESM.gif]

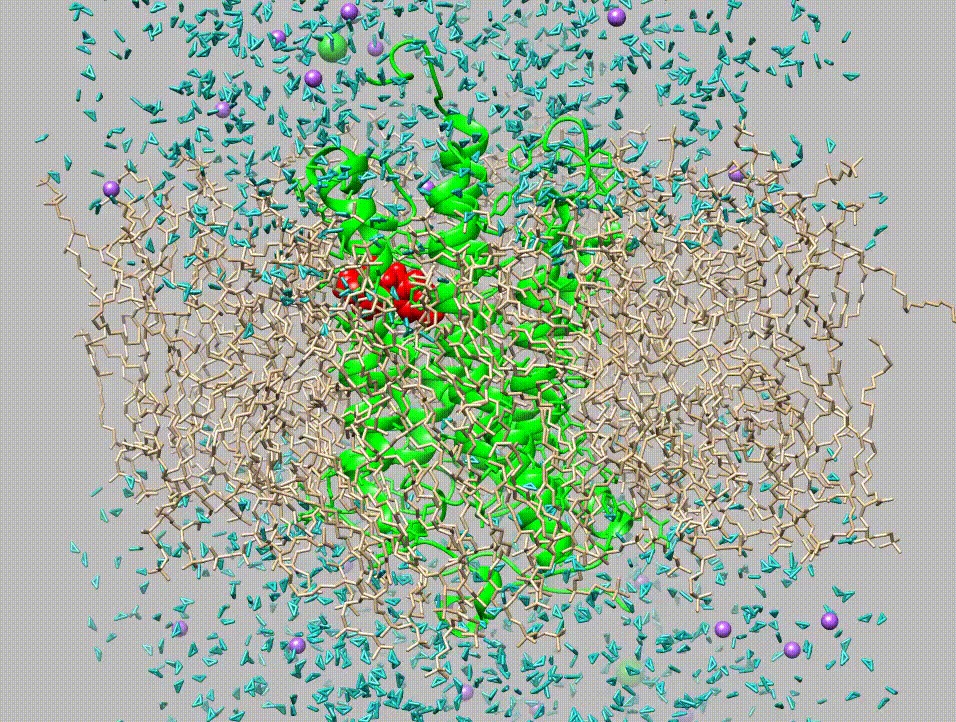

Supplement: Supplementary file 4 — Supplementary Movie 3 [file 41538_2021_114_MOESM4_ESM.gif]

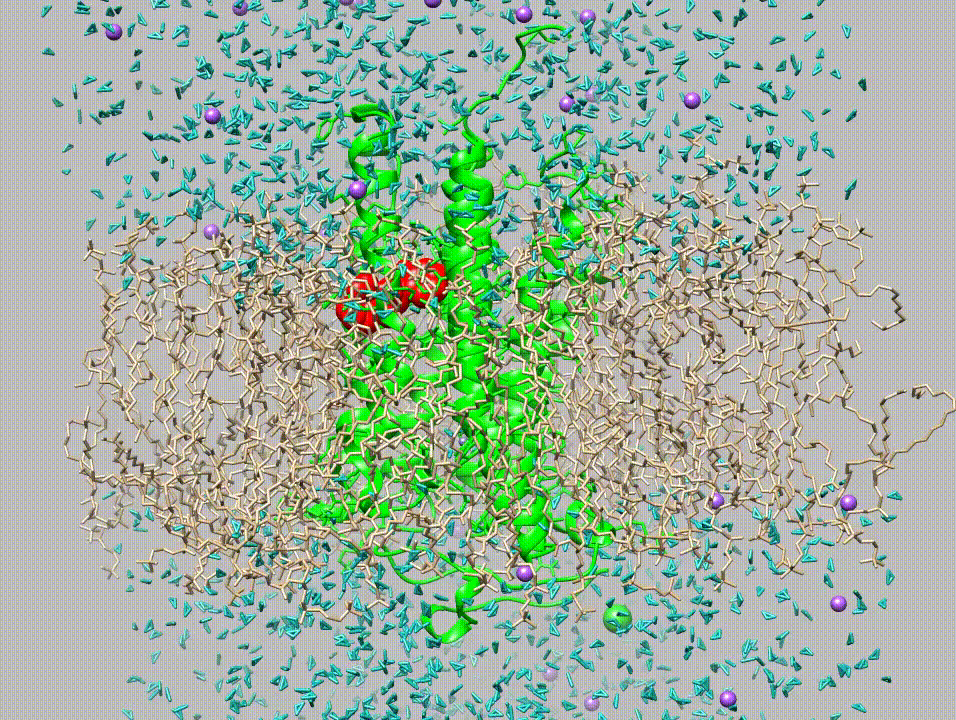

Supplement: Supplementary file 5 — Supplementary Movie 4 [file 41538_2021_114_MOESM5_ESM.gif]

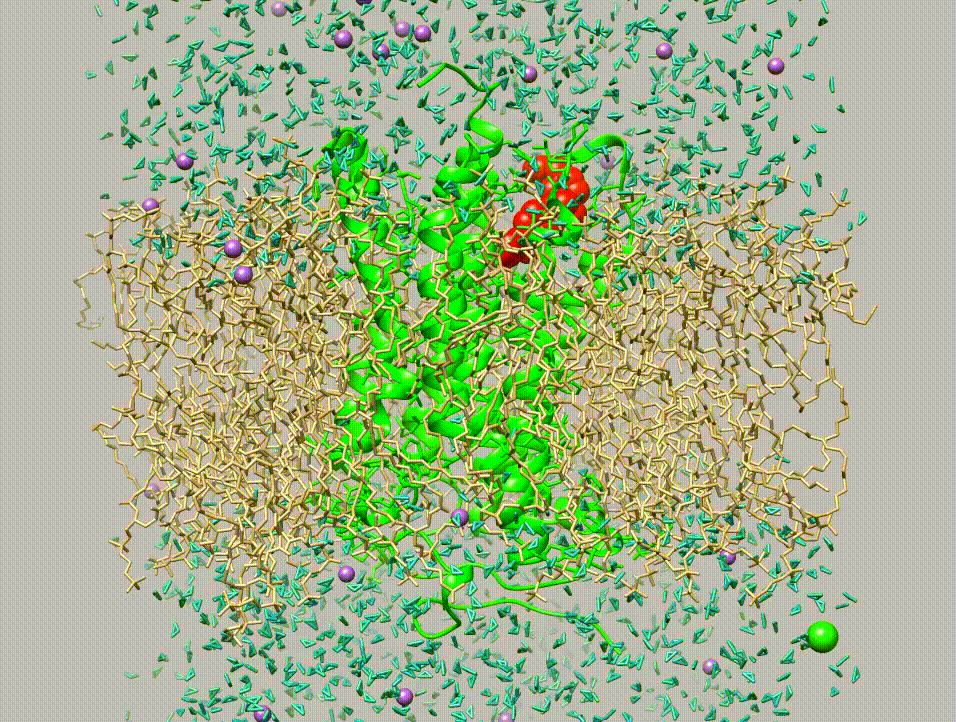

Supplement: Supplementary file 6 — Supplementary Movie 5 [file 41538_2021_114_MOESM6_ESM.gif]

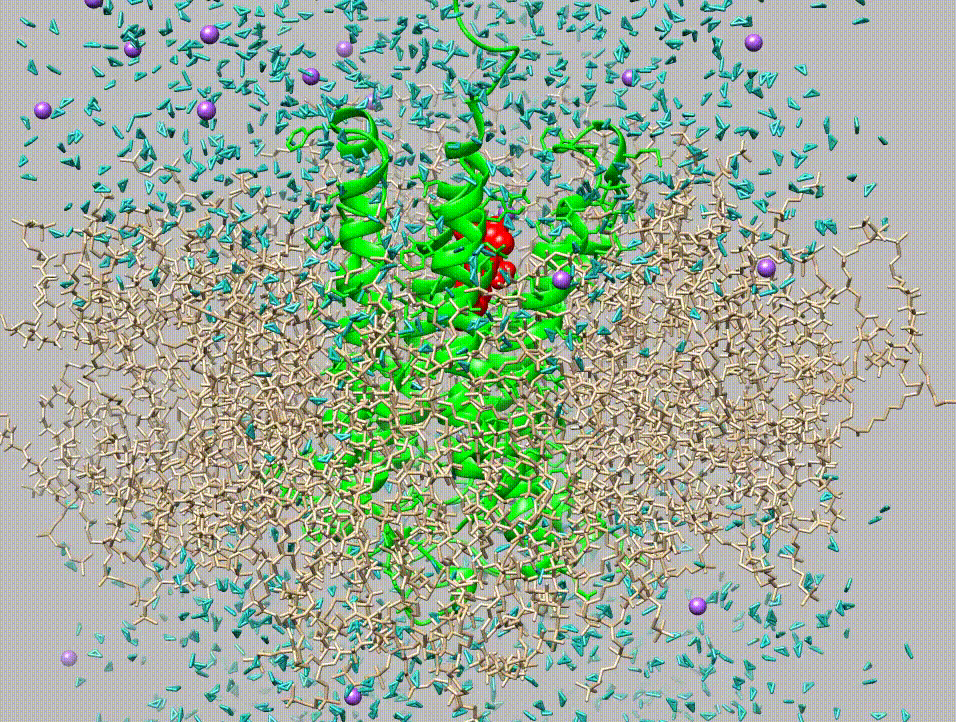

Supplement: Supplementary file 7 — Supplementary Movie 6 [file 41538_2021_114_MOESM7_ESM.gif]

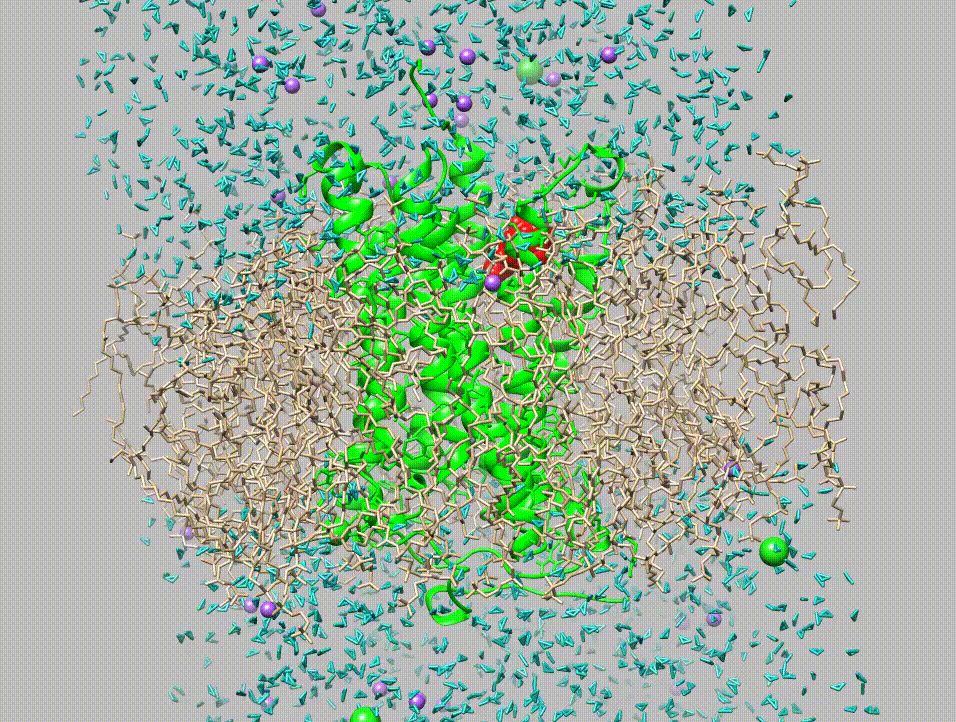

Supplement: Supplementary file 9 — Supplementary Movie 8 [file 41538_2021_114_MOESM9_ESM.gif]
