# Supplementary material for: In vitro and in silico characterization of adiponectin-receptor agonist dipeptides
Source: NPJ Sci Food. 2021 Nov 12;5:29. doi: 10.1038/s41538-021-00114-2 (PMC8589863; doi:10.1038/s41538-021-00114-2)
Supplement: Supplementary file 10 — Reporting Summary [file 41538_2021_114_MOESM10_ESM.pdf]

nature portfolio

Corresponding author(s): Toshiro Matsui NPJSCIFOOD-00406R

Last updated by author(s): Oct 11, 2021

Reporting Summary

Nature Portfolio wishes to improve the reproducibility of the work that we publish. This form provides structure for consistency and transparency in reporting. For further information on Nature Portfolio policies, see our Editorial Policies and the Editorial Policy Checklist.

Statistics

For all statistical analyses, confirm that the following items are present in the figure legend, table legend, main text, or Methods section.

n/a	Confirmed	
☐	☒	The exact sample size (n) for each experimental group/condition, given as a discrete number and unit of measurement
☐	☒	A statement on whether measurements were taken from distinct samples or whether the same sample was measured repeatedly
☐	☒	The statistical test(s) used AND whether they are one- or two-sided<br>*Only common tests should be described solely by name; describe more complex techniques in the Methods section.*
☒	☐	A description of all covariates tested
☐	☒	A description of any assumptions or corrections, such as tests of normality and adjustment for multiple comparisons
☐	☒	A full description of the statistical parameters including central tendency (e.g. means) or other basic estimates (e.g. regression coefficient) AND variation (e.g. standard deviation) or associated estimates of uncertainty (e.g. confidence intervals)
☐	☒	For null hypothesis testing, the test statistic (e.g. F, t, r) with confidence intervals, effect sizes, degrees of freedom and P value noted<br>*Give P values as exact values whenever suitable.*
☒	☐	For Bayesian analysis, information on the choice of priors and Markov chain Monte Carlo settings
☒	☐	For hierarchical and complex designs, identification of the appropriate level for tests and full reporting of outcomes
☒	☐	Estimates of effect sizes (e.g. Cohen's d, Pearson's r), indicating how they were calculated

Our web collection on statistics for biologists contains articles on many of the points above.

Software and code

Policy information about availability of computer code

Data collection	The following softwares were used for in silico analysis as described in the Methods section: Gaussian ver. 16A. 03, AMBER ver. 18, UCSF Chimera ver. 1.14, PyMOL ver. 2.3.4, MODELLER ver. 9.23, ClustalW ver. 2.1, and AutoDock Tools ver. 1.5.6.
Data analysis	All analyses were performed using GraphPad Prism ver. 5. 0.

For manuscripts utilizing custom algorithms or software that are central to the research but not yet described in published literature, software must be made available to editors and reviewers. We strongly encourage code deposition in a community repository (e.g. GitHub). See the Nature Portfolio guidelines for submitting code & software for further information.

Data

Policy information about availability of data

All manuscripts must include a data availability statement. This statement should provide the following information, where applicable:

- Accession codes, unique identifiers, or web links for publicly available datasets
- A description of any restrictions on data availability
- For clinical datasets or third party data, please ensure that the statement adheres to our policy

The data supporting the findings reported herein are available on reasonable request from the corresponding author.

Field-specific reporting

Please select the one below that is the best fit for your research. If you are not sure, read the appropriate sections before making your selection.

☒ Life sciences ☐ Behavioural & social sciences ☐ Ecological, evolutionary & environmental sciences

For a reference copy of the document with all sections, see nature.com/documents/nr-reporting-summary-flat.pdf

Life sciences study design

All studies must disclose on these points even when the disclosure is negative.

Sample size	Sample sizes were chosen according to the standards of the filed (at least three independent replications for each condition), which gave sufficient statistics.
Data exclusions	No data were excluded from analysis.
Replication	All experiments were done at least three independent replications and all attempts of replication were successful and gave similar results.
Randomization	No randomization was necessary for this study because investigators were comparing designed samples under well controlled conditions. No human or animal subjects were used in the study.
Blinding	Blinding is not applicable to this study since no individual was involved in group allocation.

Reporting for specific materials, systems and methods

We require information from authors about some types of materials, experimental systems and methods used in many studies. Here, indicate whether each material, system or method listed is relevant to your study. If you are not sure if a list item applies to your research, read the appropriate section before selecting a response.

Materials & experimental systems

n/a	Involved in the study	
☐	☒	Antibodies
☐	☒	Eukaryotic cell lines
☒	☐	Palaeontology and archaeology
☒	☐	Animals and other organisms
☒	☐	Human research participants
☒	☐	Clinical data
☒	☐	Dual use research of concern

Methods

n/a	Involved in the study	
☒	☐	ChIP-seq
☒	☐	Flow cytometry
☒	☐	MRI-based neuroimaging

Antibodies

Antibodies used	antibody for AdipoR1 (Abcam, Cat.# ab126611, Lot# GR277137-17)<br>antibody for AMPK (Merck Millipore, Cat.# 07-350, Lot# 2922422)<br>antibody for p-AMPK (Merck Millipore, Cat.# 07-681, Lot# 2901522)<br>antibody for Glut4 (Cell Signaling Technology, Cat.# 2213S, Lot# 7)
Validation	antibody for AdipoR1 : https://www.abcam.co.jp/adipor1-antibody-epr6626-ab126611.html<br>antibody for AMPK : https://www.merckmillipore.com/JP/en/product/Anti-AMPK-1-Antibody,MM_NF-07-350<br>antibody for p-AMPK : https://www.merckmillipore.com/JP/en/product/Anti-phospho-AMPK-Thr172-Antibody,MM_NF-07-681?ReferrerURL=https%3A%2F%2Fwww.google.com%2F<br>antibody for Glut4 : https://www.cellsignal.com/products/primary-antibodies/glut4-1f8-mouse-mab/2213

Eukaryotic cell lines

Policy information about cell lines

Cell line source(s)	Rat skeletal muscle L6 myoblasts (cell numer: JCRB9081, Lot# 05152017), were obtained from the Japanese Collection of Research Bioresources (JCRB Cell Bank, Osaka, Japan).
Authentication	None of the cell line used was authenticated.
Mycoplasma contamination	The cell line tested negative for mycoplasma contamination.

Commonly misidentified lines<br>(See ICLAC register)	No commonly misidentified cell line was used.
